# *Shewanella oneidensis* employs both β-lactam resistance and tolerance controlled by the PghKR system

**DOI:** 10.1128/mbio.01515-26

**Published:** 2026-09-14

**Authors:** Jingxiao Cai, Yanqun Liang, Yu Chen, Xinyu Weng, Yiwei Hu, Min Wang, Qiu Meng, Zhiliang Yu, Jianhua Yin

**Affiliations:** 1 College of Biotechnology and Bioengineering, Zhejiang University of Technology 12624, Hangzhou, China; 2 State Key Laboratory of Green Chemical Synthesis and Conversion, Zhejiang University of Technology 12624, Huzhou, China; Universite de Geneve, Geneva, Switzerland

**Keywords:** antibiotic resistance, antibiotic tolerance, two-component systems, beta-lactams, bacterial cell wall

## Abstract

Antibiotic tolerance enables bacterial survival during antibiotic exposure and can promote the evolution of resistance, thereby threatening therapeutic efficacy. However, the molecular mechanisms underlying tolerance and its relationship with resistance remain incompletely understood. In this study, we show that a class D β-lactamase-deficient strain of *Shewanella oneidensis* (∆*blaA*) is highly susceptible to β-lactams, but retains the ability to survive lethal antibiotic concentrations. Under β-lactam stress, ∆*blaA* cells form reversible spheroplasts that revert to rod-shaped morphology and resume growth upon antibiotic removal, confirming a tolerance phenotype. Genetic analysis revealed that this tolerance depends on the peptidoglycan damage-responsive two-component system PghKR, which positively regulates the marine sortase target protein SO2195. Mechanistically, SO2195 preserves cell envelope integrity, thereby preventing spheroplast swelling and lysis under cell wall stress. SO2195 homologs are widely distributed across multiple bacterial classes and are consistently located adjacent to a sortase gene, suggesting a conserved functional module. The PghKR-mediated regulatory pathway also governs tolerance to other cell wall-acting antibiotics, such as D-cycloserine. Together with our previous findings that PghKR controls *blaA*-mediated β-lactam resistance, these results establish PghKR as a central regulatory hub that coordinately controls both resistance and tolerance, allowing bacteria to dynamically balance distinct survival strategies under bactericidal antibiotic stress.

**IMPORTANCE:**

Antibiotic treatment failure is attributed not only to resistance, where bacteria grow in the presence of drugs, but also to tolerance, defined as the ability to survive bactericidal antibiotics without a change in minimum inhibitory concentration (MIC) and to resume growth upon drug removal. Whether a single bacterial population can concurrently employ and coordinate both strategies remains unclear. Such a scenario could challenge widely used combination therapies, such as those combining β-lactams with β-lactamase inhibitors. Here, using the intrinsically β-lactam-resistant bacterium *Shewanella oneidensis*, we show that bacteria can indeed employ both resistance and tolerance, and that both are controlled by a single regulatory system. Remarkably, even when primary β-lactamase-mediated resistance is abolished by genetic deletion, a dedicated tolerance pathway sustains bacterial survival. These findings reveal an integrated bacterial defense strategy and highlight the need to target both resistance and tolerance to overcome antibiotic treatment failure.

## INTRODUCTION

Antibiotics are widely used in clinical practice to treat bacterial infections due to their ability to kill or inhibit bacterial growth. However, bacteria have evolved diverse strategies to overcome antibiotic treatment, often leading to therapeutic failure. The most extensively studied strategy is antibiotic resistance, which enables bacteria to grow in the presence of antibiotics (1, 2). In recent years, increasing attention has been paid to the phenomenon of antibiotic tolerance, defined as the ability of a bacterial population to survive extended exposure to bactericidal antibiotics without an increase in the minimum inhibitory concentration (MIC). Tolerant cells remain viable during bactericidal antibiotic exposure but resume growth once the antibiotic is removed (3, 4). Importantly, growing evidence suggests that tolerance can increase the likelihood of acquiring resistance mutations, a pattern observed in pathogens such as *Pseudomonas aeruginosa*, enteropathogenic *Escherichia coli*, *Edwardsiella tarda*, and *Staphylococcus aureus*, as well as in non-pathogenic *E. coli* K12 (5–10). However, it remains unclear whether bacteria that already possess intrinsic antibiotic resistance also harbor concurrent tolerance mechanisms.

Cell wall-acting antibiotics (CWAs), which target the biosynthesis of essential cell wall components, represent one of the most important classes of antibiotics in clinical use (11). This class includes β-lactams (e.g., penicillins and carbapenems) and other inhibitors, such as D-cycloserine. Several gram-negative pathogens, including *P. aeruginosa*, *Vibrio cholerae*, and *Enterobacter cloacae*, exhibit high tolerance to bactericidal CWAs, particularly to β-lactams (11–14). The mechanisms underlying this tolerance are multifaceted and often interrelated. For instance, one contributing factor to β-lactam tolerance is reduced growth rates. Slow-growing or non-dividing cells (e.g., in stationary phase, under nutrient limitation, or in biofilms) are typically more tolerant. In addition, production of the alarmone (p)ppGpp, which triggers growth arrest, has been associated with CWA tolerance in both gram-negative and gram-positive bacteria. Morphologically, β-lactam tolerance can be mediated through the formation of spheroplasts, cells that lack detectable peptidoglycan and do not divide, thereby entering a non-growing state that permits survival under CWA stress (12, 15–17).

Two-component systems (TCSs) are well-known for sensing environmental signals and regulating adaptive responses. To date, however, only two TCSs, PhoPQ in *Enterobacterales* and VxrAB in *V. cholerae*, have been clearly shown to regulate β-lactam tolerance. Both are indirectly activated by β-lactam antibiotics but function through different mechanisms. PhoPQ enhances outer membrane stability, whereas VxrAB upregulates peptidoglycan synthesis genes and downregulates iron acquisition systems (11, 13, 14, 18). Given the diversity of bacterial species and the complexity of cell envelope stress responses, it is likely that additional TCSs contribute to antibiotic tolerance in other organisms.

*Shewanella* species are environmentally widespread, particularly in aquatic habitats, and have also been isolated from clinical settings. They exhibit considerable metabolic versatility and intrinsic resistance to various toxic compounds (19–21). Members of this genus, such as *S. algae*, are recognized as opportunistic pathogens associated with human infections, including diarrhea, otitis media, soft-tissue infections, septicemia, and hepatobiliary diseases (22–24). Notably, *Shewanella* species are regarded as natural reservoirs of antibiotic resistance genes, including those conferring resistance to β-lactam antibiotics, polymyxins, and quinolones (24–28). As a representative species, *S. oneidensis* produces a carbapenem-hydrolyzing class D β-lactamase, BlaA (OXA-54), a putative progenitor of the clinically prevalent OXA-48-type enzymes (29, 30). We previously showed that BlaA confers inducible β-lactam resistance, and its loss (∆*blaA*) results in marked hypersensitivity (31–34). More recently, we identified a novel TCS, PghKR, that senses peptidoglycan damage and is responsible for regulating *blaA* expression (35). This discovery positions *S. oneidensis* as a relevant model to investigate how bacteria coordinate survival strategies under cell wall stress.

In this study, we demonstrate that *S. oneidensis* exhibits tolerance to lethal concentrations of β-lactam antibiotics even when its primary resistance determinant BlaA is absent. This survival is mediated by reversible spheroplast formation and is governed by the PghKR system. We further identify a PghKR-regulated sortase-associated gene, *SO2195*, as a key contributor to β-lactam tolerance. The same regulatory pathway also confers tolerance to other CWAs, revealing a coordinated system that enables *S. oneidensis* to survive diverse cell envelope stresses.

## RESULTS

### The β-lactamase-deficient *S. oneidensis* strain ∆*blaA* exhibits tolerance to β-lactam antibiotics

*S. oneidensis* is intrinsically resistant to β-lactam antibiotics, such as ampicillin and carbenicillin, primarily due to the production of the β-lactamase BlaA that hydrolyzes these antibiotics (31–34). Consequently, the β-lactamase-deficient strain ∆*blaA* is highly sensitive, with an MIC of 0.5 μg/mL. Interestingly, when ∆*blaA* cells were first exposed to a lethal concentration of ampicillin (50 μg/mL) for 2 h and then washed to remove the antibiotic, they formed colonies on antibiotic-free LB plates (Fig. S1). In contrast, *E. coli* K-12 MG1655, a known β-lactam-sensitive and non-tolerant bacterium, failed to regrow under the same conditions (Fig. S1) (12, 13). These observations suggest that a subpopulation of ∆*blaA* cells remains viable even after exposure to lethal concentrations of ampicillin.

**Fig 1:**
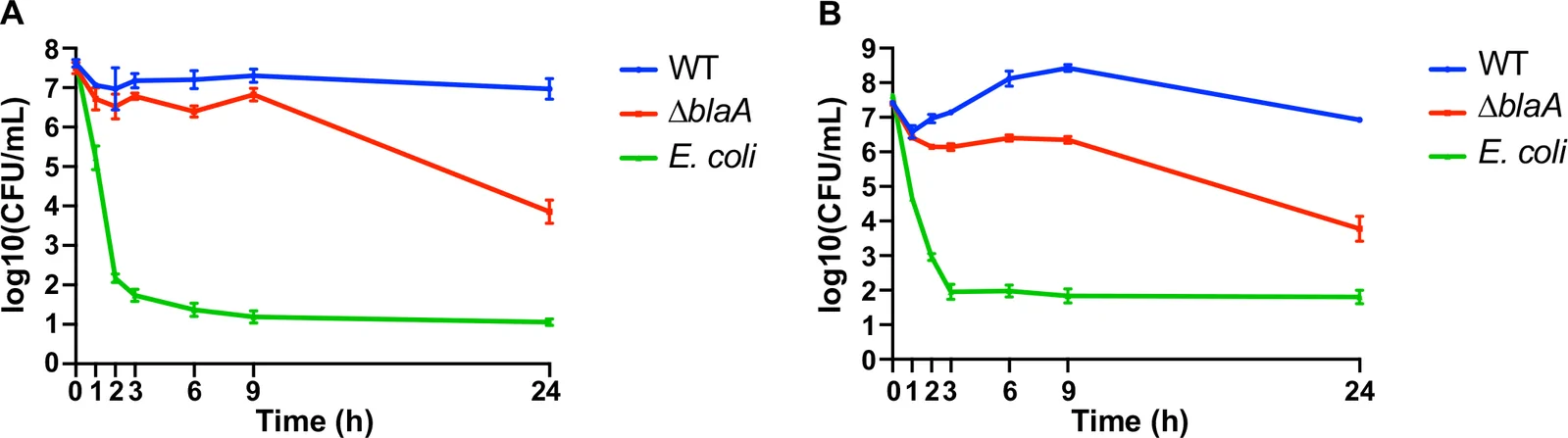
The *S. oneidensis* ∆*blaA* strain is tolerant to β-lactam antibiotics. Time-dependent killing curves of the indicated strains in the presence of 20 μg/mL ampicillin (**A**) or 256 μg/mL carbenicillin (**B**), both at concentrations exceeding their respective MICs (Table S1). Exponential-phase cultures (OD_600_ of ~0.1) were treated with antibiotics, and CFU/mL was determined at the indicated time points. Data are shown as mean ± S.D. of log_10_-transformed values from three biological replicates (*n* = 3).

To test this hypothesis, *S. oneidensis* wild type (WT), ∆*blaA*, and *E. coli* were subjected to time-killing assays with ampicillin or carbenicillin at concentrations exceeding their respective MICs (Table S1). As expected, *E. coli* was rapidly killed, with colony-forming units (CFU) decreasing by 10^5^- to 10^6^-fold within 3 h (Fig. 1A and B). In contrast, the resistant *S. oneidensis* WT survived under these conditions, although ampicillin limited its growth due to partial lysis as observed in our previous study (31) (Fig. 1; Fig. S2). Meanwhile, ∆*blaA* showed initial survival, followed by a gradual decline in viability. Notably, even after 24 h of antibiotic exposure, a substantial number of ∆*blaA* cells remained viable (approximately 10^4^ CFU/mL with ampicillin and 10^3^ CFU/mL with carbenicillin) (Fig. 1A and B). These results demonstrate that ∆*blaA* exhibits tolerance to β-lactams, revealing that *S. oneidensis* possesses both resistance and tolerance mechanisms against this class of antibiotics.

### β-Lactam tolerance of *S. oneidensis* is mediated by reversible spheroplast formation

**Fig 2:**
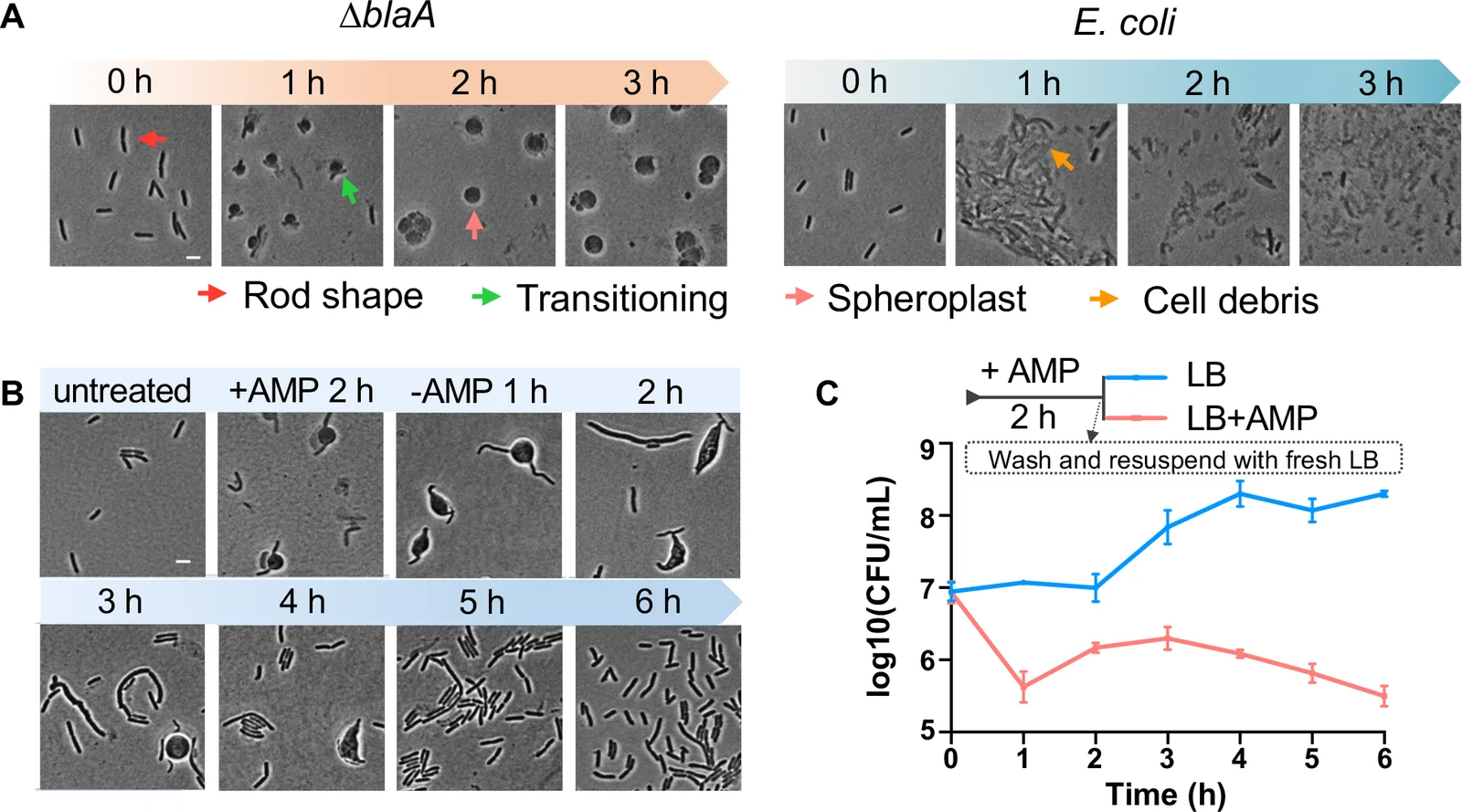
The ∆*blaA* mutant forms tolerant spheroplasts upon β-lactam exposure. (**A**) Cell morphology of *S. oneidensis* ∆*blaA* and *E. coli* MG1655 upon exposure to 256 μg/mL carbenicillin for the indicated times. (**B**) Cell morphology of ∆*blaA* treated with 20 μg/mL ampicillin (+AMP) for 2 h, followed by antibiotic removal (−AMP) and incubation in fresh medium for 6 h. (**C**) Recovery of ∆*blaA* cells after antibiotic removal. Cells were treated with 20 μg/mL ampicillin for 2 h, washed, and resuspended in fresh LB with or without ampicillin. Viability (CFU/mL) was monitored every hour. Data are shown as mean ± S.D. of log_10_-transformed values from three biological replicates (*n* = 3). Scale bar, 2.5 μm.

Exposure to β-lactam antibiotics can induce many gram-negative bacteria to form non-dividing, cell wall-deficient spheroplasts (11). We observed a similar morphological shift in *S. oneidensis* ∆*blaA* upon treatment with carbenicillin. As shown in Fig. 2A, untreated cells of both *E. coli* and *S. oneidensis* ∆*blaA* displayed typical rod-shaped morphology. Following treatment with carbenicillin, the two strains responded differently. *E. coli* cells lysed rapidly, with nearly all cells lysed within 2 h. In contrast, ∆*blaA* cells first bulged and enlarged at mid-cell within 1 h and then transitioned into spheroplasts by 2 h. These results indicate that carbenicillin induces spheroplast formation in *S. oneidensis* ∆*blaA*, which prevents cell lysis and promotes survival, thereby explaining the β-lactam tolerance phenotype.

We next investigated whether these spheroplasts could revert to normal morphology and resume growth. ∆*blaA* cells were treated with ampicillin for 2 h, washed to remove the antibiotic, and then monitored for morphological recovery and viability (CFU/mL). Upon ampicillin exposure, rod-shaped cells enlarged and transformed into spheroplasts. After antibiotic removal, these spherical cells transitioned through irregular and filamentous shapes within 3 h, and most cells regained the typical rod-shaped morphology by 3–4 h (Fig. 2B). Concomitantly, CFU values remained stable for the first 2 h post-washout but increased markedly thereafter (Fig. 2C). This recovery in viability corresponded closely with the morphological observations, in which a substantial population of rod-shaped cells reappeared after 3 h of incubation (Fig. 2B). Together, these findings demonstrate that β-lactam tolerance in ∆*blaA* is mediated by reversible spheroplast formation, which allows a subpopulation of cells to survive antibiotic exposure and resume growth once the stress is removed.

### The two-component system PghKR is involved in β-lactam tolerance

**Fig 3:**
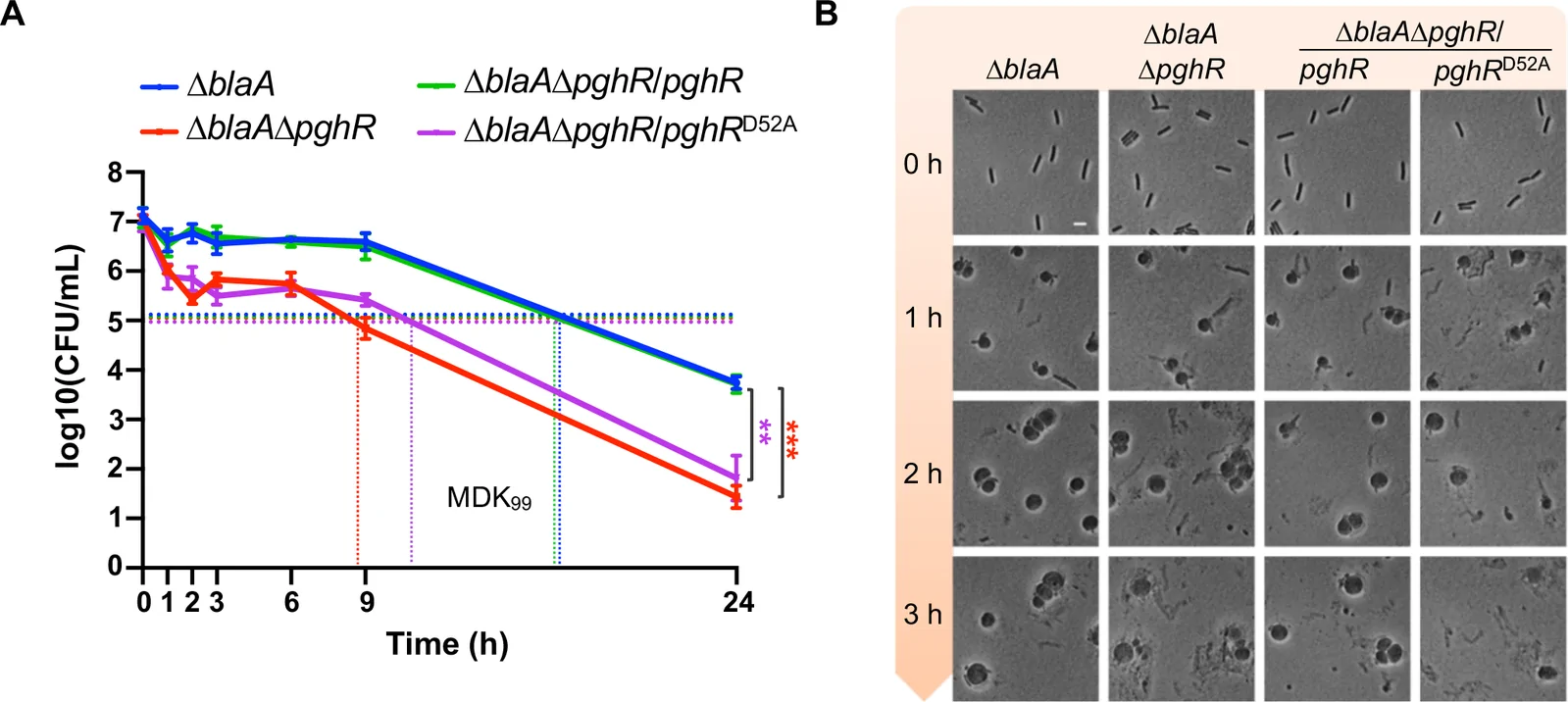
The two-component system PghKR is required for β-lactam tolerance. (**A**) Time-dependent killing curves of the indicated strains in the presence of 256 μg/mL carbenicillin, at concentrations exceeding the MICs of all *blaA* deletion mutants (see Table S1). CFU/mL was measured at the indicated time points. The vertical dashed line indicates the minimum duration required to kill 99% of the bacterial population (MDK_99_). Data are shown as mean ± S.D. of log_10_-transformed values from three biological replicates (*n* = 3). (**B**) Cell morphology of the indicated strains treated with 256 μg/mL carbenicillin at 0, 1, 2, and 3 h. Scale bar, 2.5 μm.

We previously reported that the novel TCS PghKR is activated by β-lactam antibiotics (36). This activation led us to hypothesize that PghKR may contribute to β-lactam tolerance. To test this, we compared the survival of the ∆*blaA* strain and a ∆*blaA*∆*pghR* double mutant challenged with carbenicillin. Time-killing curves revealed significantly reduced viability of the double mutant relative to ∆*blaA*. After 2 h of carbenicillin exposure, viable ∆*blaA*∆*pghR* cells decreased approximately 10-fold compared to ∆*blaA*, and the reduction reached nearly 250-fold by 24 h (Fig. 3A). Complementation with *pghR* (∆*blaA*∆*pghR*/*pghR*) restored survival to ∆*blaA* levels (Fig. 3A), confirming that PghR is required for full carbenicillin tolerance.

In canonical TCSs, the histidine kinase is auto-phosphorylated at a conserved histidine residue and subsequently transfers the phosphoryl group to a conserved aspartate residue on its cognate response regulator (37). For PghKR, the conserved aspartate 52 (Asp52) of PghR is the identified phosphorylation site (35). To determine whether β-lactam tolerance depends on phosphorylation of PghKR, we constructed an unphosphorylated PghR variant in which Asp52 was substituted with alanine (PghR^D52A^), and introduced it into the ∆*blaA*∆*pghR* background. This strain (∆*blaA*∆*pghR*/*pghR*^D52A^) failed to restore tolerance (Fig. 3A), indicating that functional phosphoryl transfer within PghKR is essential. To further confirm, we compared the minimum duration required to kill 99% of the bacterial population (MDK_99_) among different strains. Strains lacking functional PghR (∆*blaA*∆*pghR* and ∆*blaA*∆*pghR*/*pghR*^D52A^) exhibited markedly shorter MDK_99_ values than strains expressing wild-type PghR (∆*blaA* and ∆*blaA*∆*pghR*/*pghR*) (Fig. 3A). These results demonstrate that phosphorylated PghR is crucial for mediating β-lactam tolerance.

We then examined whether PghKR affects spheroplast formation. After 3 h of carbenicillin exposure, ∆*blaA*∆*pghR* and ∆*blaA*∆*pghR*/*pghR*^D52A^ strains exhibited fewer spherical cells and more cell debris compared to ∆*blaA* (Fig. 3B), suggesting that PghR promotes the stabilization of spheroplasts under β-lactam stress.

### PghKR-mediated β-lactam tolerance is related to SO2195

**Fig 4:**
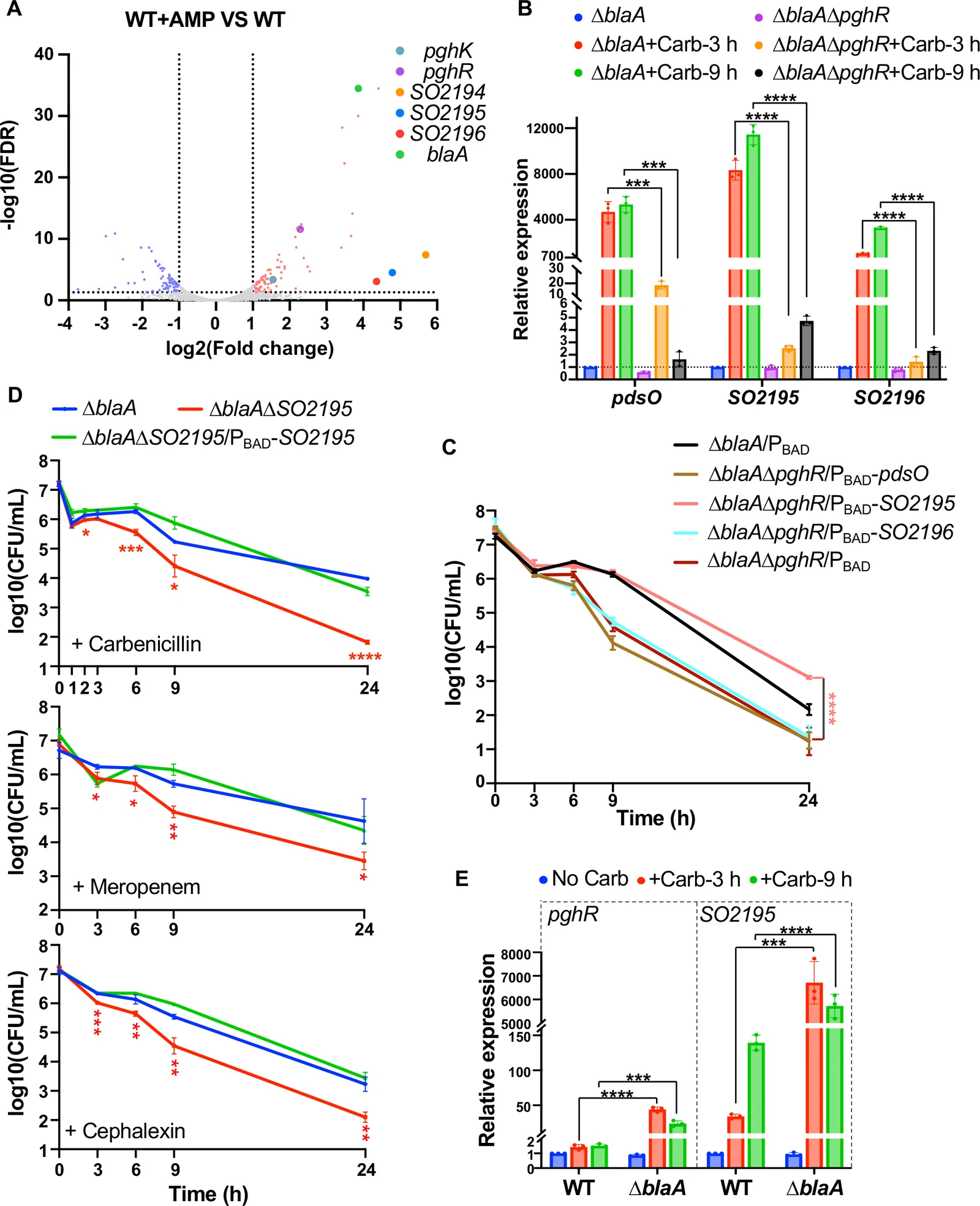
PghKR-mediated β-lactam tolerance is related to *SO2195*. (**A**) Volcano plot of transcriptomic differences between WT cells treated with or without 100 μg/mL AMP. Genes of interest are highlighted. (**B**) Relative transcriptional expression of *pdsO*, *SO2195*, and *SO2196* in ∆*blaA* and ∆*blaA*∆*pghR*, with or without 256 μg/mL carbenicillin (Carb) treatment for the indicated times, as determined by quantitative real-time reverse transcription PCR (qRT-PCR). Expression was normalized to that of untreated Δ*blaA* cells, using 16S rRNA as the internal reference. (**C**) Time-dependent killing curves of the indicated strains in the presence of 256 μg/mL carbenicillin. (**D**) Time-dependent killing curves of the indicated strains in the presence of carbenicillin, meropenem, and cephalexin at concentrations exceeding their respective MICs (see Table S1 for details). (**E**) Relative transcriptional expression of *pghR* and *SO2195* in WT and ∆*blaA*, with or without 256 μg/mL Carb treatment for the indicated times. Expression was normalized to that of untreated WT cells, using 16S rRNA as the internal reference. Arabinose was added at 0.01% (wt/vol) where indicated. Data in panels C and D are shown as mean ± S.D. of log_10_-transformed values from three biological replicates (*n* = 3).

Previous RNA-seq analysis revealed that ampicillin treatment activates several PghR regulon members, including *relV* [encoding a (p)ppGpp synthetase], *creD* (encoding a cell envelope integrity protein), *phoPQ* (encoding a two-component system), and a gene cluster comprising *SO2194* (*pdsO*), *SO2195*, and *SO2196* (Fig. 4A) (35). We focused on the *pdsO-SO2195-SO2196* cluster because it forms an operon located immediately adjacent to *pghKR* on the chromosome. NCBI annotations indicate that *pdsO* encodes a sortase-associated OmpA-like protein, *SO2195* encodes a marine proteobacterial sortase target protein, and *SO2196* encodes a Class GN sortase or marine sortase. Based on these predicted functions and its genomic location, we hypothesized that this cluster may contribute to antibiotic tolerance.

To test this possibility, we first examined whether *pdsO*, *SO2195*, and *SO2196* were induced by carbenicillin in a PghR-dependent manner. As shown in Fig. 4B, all three genes were significantly induced in ∆*blaA* after 3 and 9 h of carbenicillin exposure, but this induction was nearly abolished in the ∆*blaA*∆*pghR* background, confirming their PghR-dependent transcriptional activation. To assess whether downregulation of these genes underlies the tolerance defect in the ∆*pghR* mutant, we performed complementation with arabinose-inducible expression plasmids. While the empty-vector control (∆*blaA*/P_BAD_) showed no effect, ectopic expression of *SO2195* (∆*blaA*∆*pghR*/P_BAD_-*SO2195*) fully rescued the survival of the double mutant under carbenicillin exposure (Fig. 4C; Fig. S3). In contrast, overexpression of other PghR regulon members (*relV*, *creD*, and *phoPQ*) in the ∆*pghR* background did not significantly restore tolerance (Fig. S4), further supporting the specific role of SO2195. Furthermore, deletion of *SO2195* in the ∆*blaA* background (∆*blaA*∆*SO2195*) led to a significant survival defect under carbenicillin stress, and this defect was fully restored by *SO2195* overexpression (∆*blaA*∆*SO2195*/P_BAD_-*SO2195*) (Fig. 4D). Arabinose used in this experiment (0.01%, wt/vol) had no effect on bacterial growth (Fig. S5), ruling out non-specific effects due to overexpression toxicity. We next tested whether the role of SO2195 extends to other β-lactam classes. Time-killing assays with meropenem (a carbapenem) and cephalexin (a cephalosporin) showed that the ∆*blaA*∆*SO2195* mutant also exhibited significantly reduced survival compared to ∆*blaA*, and this defect was again rescued by *SO2195* overexpression (Fig. 4D).

**Fig 5:**
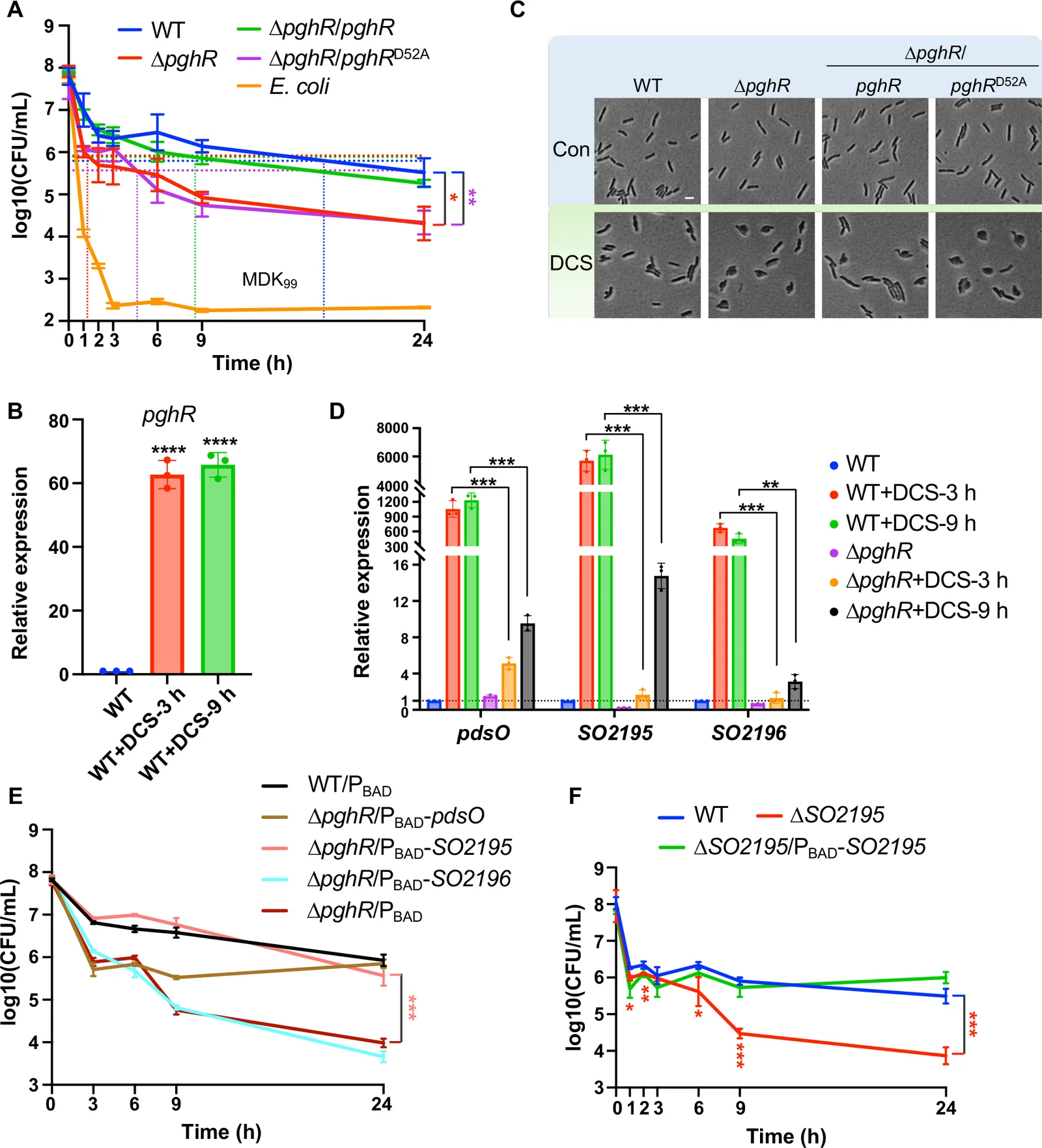
PghKR promotes D-cycloserine tolerance through *SO2195*. (**A**) Time-dependent killing curves of the indicated strains exposed to 120 μg/mL D-cycloserine (DCS), at concentrations exceeding their respective MICs (see Table S1). The vertical dashed line indicates MDK_99_. (**B, D**) Relative transcriptional expression of the indicated genes treated with DCS for 3 or 9 h, as determined by qRT-PCR. Expression was normalized to that of untreated WT cells, using 16S rRNA as the internal reference. (**C**) Cell morphology of the indicated strains after DCS exposure. Scale bar, 2.5 μm. (**E**) Complementation of DCS tolerance in the Δ*pghR* background by overexpression of *pdsO*, *SO2195*, and *SO2196*. (**F**) Time-dependent killing curves of WT, ∆*SO2195*, and ∆*SO2195*/P_BAD_-*SO2195* in the presence of DCS. Arabinose was added at 0.01% (wt/vol) where indicated. Data in panels A, E, and F are shown as mean ± S.D. of log_10_-transformed values from three biological replicates (*n* = 3).

Given the key role of PghKR in antibiotic stress response, we hypothesized that it might be hyper-activated in ∆*blaA* to compensate for the lack of β-lactamase. Indeed, transcriptional analysis showed that both *pghR* and *SO2195* were more strongly induced by carbenicillin in ∆*blaA* than in the WT, with *SO2195* induction being particularly pronounced (Fig. 4E). This indicates that PghKR and its target SO2195 play an enhanced role in sustaining survival under β-lactam stress in the absence of BlaA.

### PghKR and its target gene *SO2195* confer tolerance to diverse CWAs

To examine whether PghKR-dependent tolerance extends beyond β-lactams, we tested its role against D-cycloserine, a CWA that inhibits both D-Ala-D-Ala ligase and alanine racemase, thereby blocking peptidoglycan biosynthesis (11). Time-killing assays showed that *E. coli* survival dropped by approximately 10^5^-fold within 3 h of D-cycloserine exposure, whereas *S. oneidensis* WT exhibited only a ~100-fold reduction after 24 h (Fig. 5A). Morphological analysis revealed that *S. oneidensis* WT gradually accumulated spheroplasts over time, whereas *E. coli* cells lysed (Fig. S6), confirming that *S. oneidensis* is tolerant to D-cycloserine.

The expression of *pghR* was strongly induced by D-cycloserine (>60-fold after 3 h; Fig. 5B). Correspondingly, the ∆*pghR* and ∆*pghR*/*pghR*^D52A^ strains exhibited an ~30-fold reduction in CFU/mL and shorter MDK_99_ values compared to the WT or complemented strain (Fig. 5A), and showed fewer intact cells (Fig. 5C). The PghR target genes *pdsO*, *SO2195*, and *SO2196* were also upregulated by D-cycloserine in a PghR-dependent manner (Fig. 5D). We tested whether overexpression of each of these genes in the ∆*pghR* background could restore D-cycloserine tolerance. Among them, only *SO2195* overexpression (∆*pghR*/P_BAD_-*SO2195*) significantly improved survival (Fig. 5E). *SO2196* overexpression had no effect, whereas *pdsO* overexpression did not enhance survival during the first 6 h but increased viability at later time points (Fig. 5E). Moreover, deletion of *SO2195* caused a pronounced survival defect under D-cycloserine stress, which was fully rescued by complementation (∆*SO2195*/P_BAD_-*SO2195*) (Fig. 5F; Fig. S5). We also tested whether overexpression of other PghR regulon members, including *relV*, *phoPQ*, and *creD*, could restore D-cycloserine tolerance in the ∆*pghR* background. None of them showed significant rescue (Fig. S4). These results demonstrate that the PghKR-SO2195 regulatory circuit is also essential for D-cycloserine tolerance.

### SO2195 maintains cell envelope integrity to stabilize spheroplasts

To investigate how SO2195 contributes to antibiotic tolerance, we first examined whether it might act as a β-lactamase. Using the nitrocefin chromogenic assay, we found that β-lactamase activity did not differ between the ∆*SO2195* or ∆*blaA*∆*SO2195* mutants and their respective parental strains (WT and ∆*blaA*) (Fig. S7). Consistently, the MICs of carbenicillin, meropenem, and cephalexin were also unchanged upon *SO2195* deletion in both backgrounds (Table S1), ruling out direct antibiotic inactivation.

**Fig 6:**
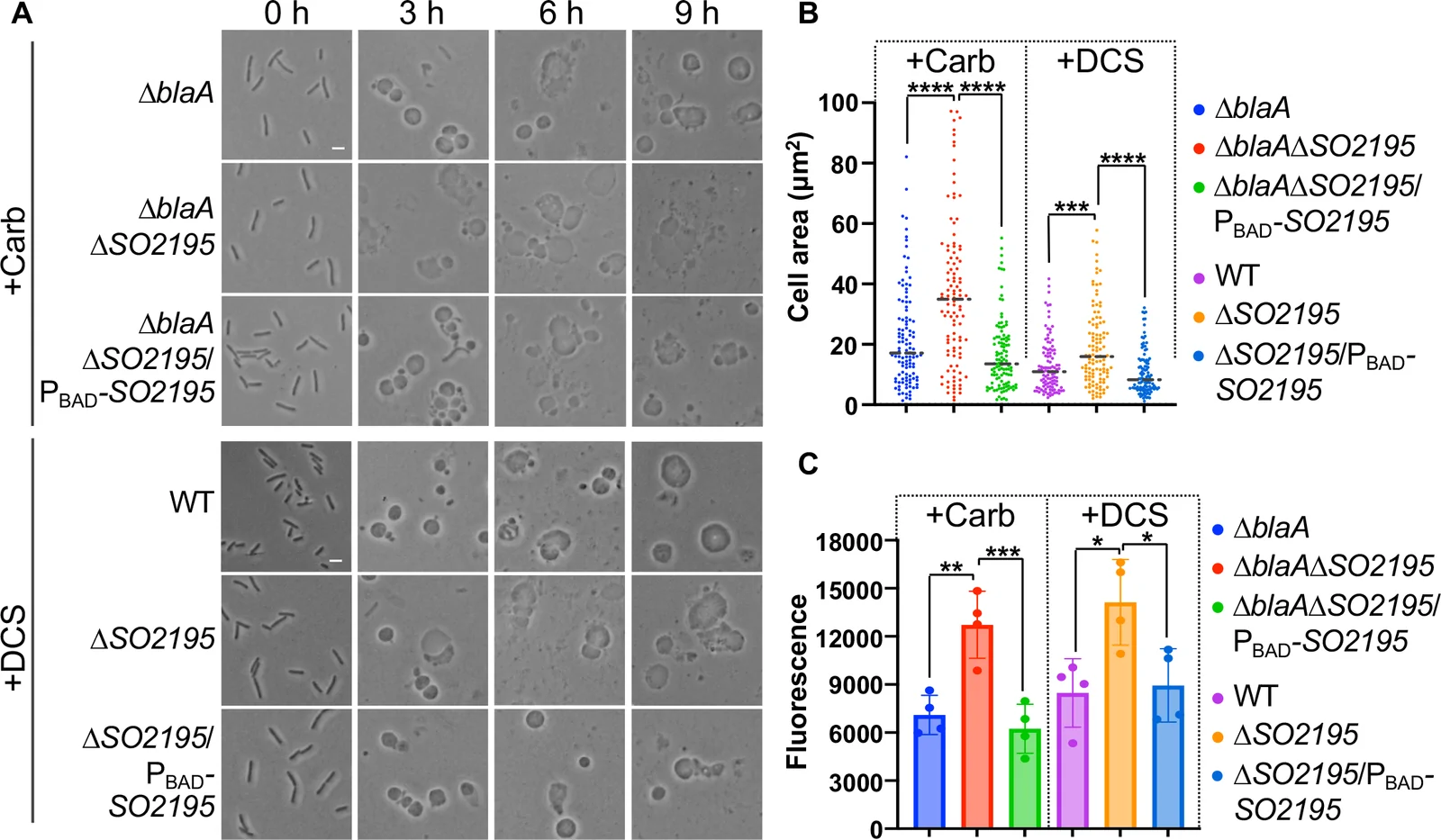
SO2195 maintains inner membrane integrity and prevents spheroplast lysis. (**A**) Cell morphology of the indicated strains treated with 256 μg/mL carbenicillin (Carb) or 120 μg/mL D-cycloserine (DCS) at the indicated time points. (**B**) Quantification of cell area from panel A at 6 h (*n* > 100). Significance was determined using the Kruskal-Wallis test. (**C**) Propidium iodide (PI) fluorescence after 6 h of treatment with carbenicillin or D-cycloserine. Significance was determined using ordinary one-way ANOVA. Arabinose was added at 0.01% (wt/vol) where indicated.

We then examined the morphology of *SO2195* deletion strains under antibiotic treatment. In the presence of carbenicillin, the ∆*blaA*∆*SO2195* mutant still formed spheroplasts, as did the ∆*SO2195* mutant under D-cycloserine treatment. These observations indicate that SO2195 is not required for spheroplast initiation. However, these spheroplasts swelled more rapidly and lysed more readily compared to those of the parental and complemented strains (Fig. 6A and B). This phenotype became more pronounced at later time points (e.g., 6 or 9 h), suggesting that SO2195 is critical for maintaining spheroplast stability regardless of the strain background.

To determine whether cell envelope integrity was compromised, we first assessed outer membrane permeability using the 1-N-phenylnaphthylamine (NPN) uptake assay. No significant difference was observed between the *SO2195* deletion mutants and their respective controls under the conditions tested (Fig. S8). We then examined inner membrane permeability using propidium iodide (PI) staining. Under antibiotic stress, PI uptake was significantly increased in *SO2195* deletion strains compared to the controls (Fig. 6C). Together, these results suggest that SO2195 contributes to cell envelope integrity, with a pronounced effect on inner membrane permeability, thereby preventing excessive spheroplast swelling and premature lysis.

### SO2195 is widely distributed across multiple bacterial lineages

**Fig 7:**
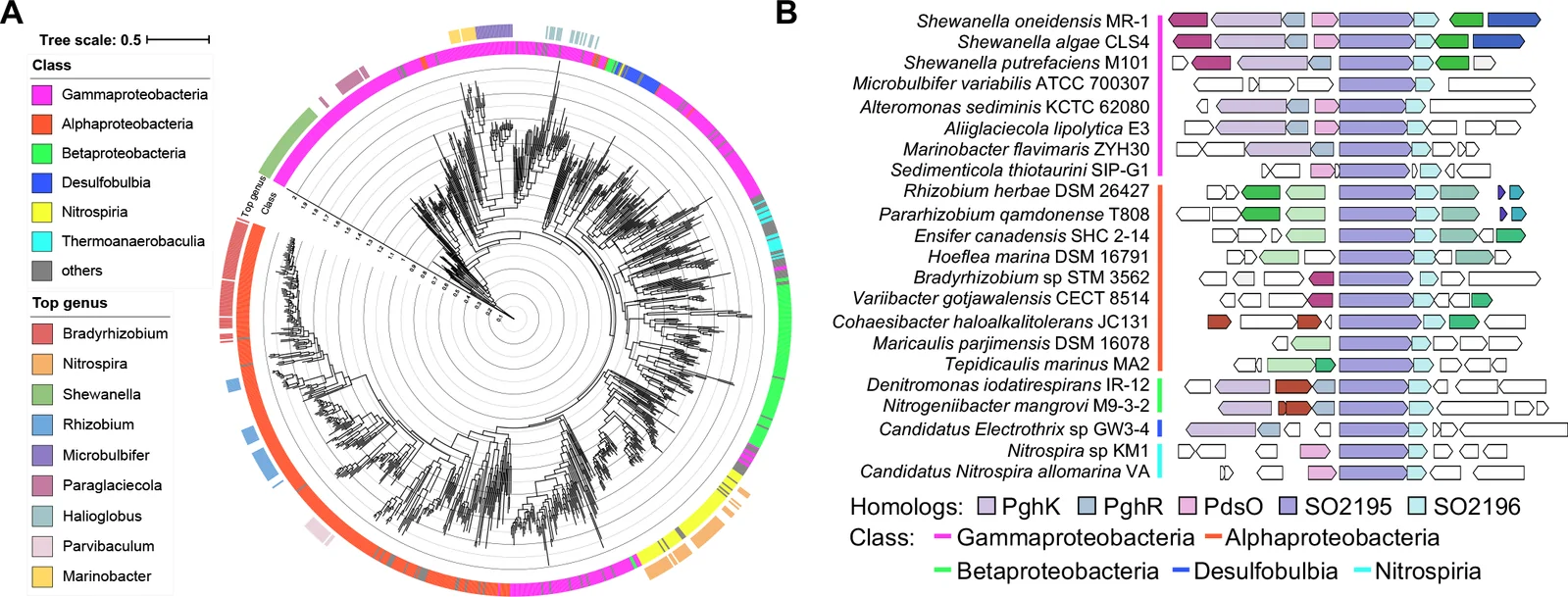
Phylogenetic analysis and genomic organization of SO2195 homologs. (**A**) Maximum likelihood tree of SO2195 homologs. Colored bars indicate the taxonomic class (inner) and genus (outer) of each homolog. (**B**) Gene neighborhood analysis of SO2195 homologs in representative strains. Genes are shown as block arrows and colored according to their predicted functions.

To assess the phylogenetic distribution of SO2195, we retrieved 1,000 homologs from the NCBI ClusteredNR database using PSI-BLAST. Phylogenetic analysis revealed that SO2195 homologs are broadly distributed across Proteobacteria (Alpha-, Beta-, and Gammaproteobacteria) and several other lineages such as Desulfobulbia and Nitrospiria (Fig. 7A), with high enrichment in genera, including *Shewanella*, *Microbulbifer*, *Bradyrhizobium*, and *Nitrospira* (Fig. 7A). NCBI Conserved Domain Database annotation indicates that SO2195 belongs to the marine_srt_targ family (TIGR03788), which contains a sortase recognition motif and a C-terminal transmembrane domain. Gene neighborhood analysis confirmed that a sortase gene is consistently located immediately downstream of SO2195 homologs in representative strains from different lineages, and this syntenic organization extends beyond Proteobacteria to lineages, such as Desulfobulbia and Nitrospiria (Fig. 7B).

## DISCUSSION

**Fig 8:**
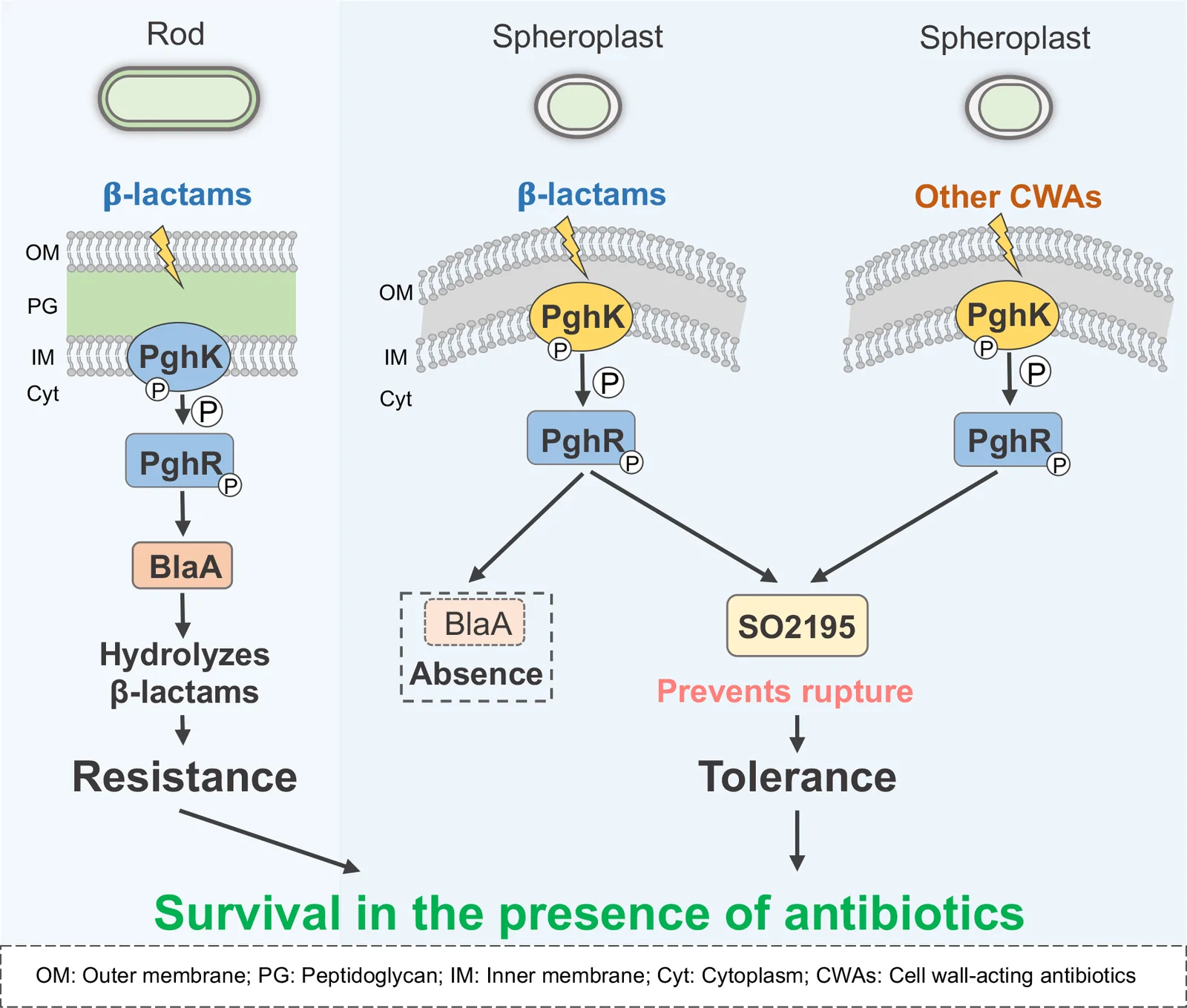
Survival strategies of *S. oneidensis* under CWA stress. Upon entry of β-lactam antibiotics into the periplasm, peptidoglycan synthesis is disrupted. This damage is sensed by the TCS PghKR. When functional BlaA β-lactamase is present, PghKR activates *blaA* expression, leading to BlaA production and drug hydrolysis, which confers β-lactam resistance. In the absence of BlaA-mediated resistance, PghKR is further activated and upregulates the expression of the sortase target protein SO2195, promoting survival via a tolerance pathway by maintaining cell envelope integrity and preventing spheroplast lysis. The PghKR-SO2195 regulatory pathway also responds to other CWAs, such as D-cycloserine, indicating a broad mechanism for coping with cell wall stress.

Bacterial tolerance to antibiotics and its contribution to the evolution of resistance are key factors underlying treatment failure. In this study, we show that *S. oneidensis* survives lethal β-lactam exposure even in the absence of its intrinsic β-lactam resistance through an active tolerance mechanism. This response is controlled by the peptidoglycan damage-responsive TCS PghKR, which is further upregulated when β-lactamase is absent and activates expression of the sortase-target protein SO2195 to enhance survival. Furthermore, PghKR and its target gene, *SO2195*, also confer tolerance to another CWA, D-cycloserine (Fig. 8). In both cases, tolerance is associated with the formation of spheroplasts. Collectively, these results uncover a coordinated survival strategy in bacteria and establish PghKR as a key regulator that integrates the response to cell wall stress.

The combination of β-lactam antibiotics with β-lactamase inhibitors represents a major therapeutic strategy against infections caused by β-lactamase-producing organisms, as it directly counteracts β-lactamase-mediated resistance (38). However, our findings reveal a potential limitation of this strategy. Even when the primary resistance enzyme BlaA is inactivated, *S. oneidensis* can still survive β-lactam exposure via tolerance (Fig. 1 and 2). Although developing effective inhibitors against OXA-type enzymes, such as BlaA, remains challenging, our results indicate that disabling resistance alone may not eliminate a bacterial population if an accompanying tolerance pathway remains functional. This observation aligns with reports of spheroplast-mediated tolerant cells isolated from patients infected with gram-negative pathogens (12, 39). Our study extends this concern beyond classical pathogens by demonstrating that an environmentally widespread, opportunistic bacterium, *S. oneidensis*, also survives high antibiotic concentrations through spheroplast formation. Moreover, similar to carbapenem-tolerant pathogens, β-lactam-tolerant *S. oneidensis* cells can revert to rod-shaped morphology and resume growth once antibiotic pressure is removed (Fig. 2), highlighting the transient and adaptive nature of this phenotype.

Resistance and tolerance to β-lactams are typically controlled by separate regulatory networks. In gram-negative bacteria, the induction of β-lactamases commonly involves dedicated regulators, such as AmpR (40–42), VbrKR (43), and BlrAB (44). In contrast, the regulators responsible for β-lactam tolerance remain less characterized, with only a few identified to date including the VxrAB system in *V. cholerae* (11, 14, 18) and the PhoPQ system in *Enterobacterales* (13). Thus, regulatory systems involved in β-lactam responses have largely been characterized as being specialized for either resistance or tolerance, but not both. In contrast, our findings reveal that the TCS PghKR in *S. oneidensis* represents an unusual example of a single regulatory system that coordinates these two survival strategies. Building on our previous work demonstrating that PghKR controls *blaA*-mediated β-lactam resistance (36), we show here that PghKR is also essential for activation of a distinct tolerance program (Fig. 3). Notably, deletion of *blaA* further elevates *pghKR* expression (Fig. 4E), indicating regulatory crosstalk between resistance and tolerance pathways. Together, these results support a model in which PghKR integrates cell wall stress signals to coordinate antibiotic resistance and tolerance.

The marine sortase gene cluster *pdsO-SO2195-SO2196* is among the genes regulated by PghKR, leading us to investigate its potential role in tolerance. Sortases are cysteine transpeptidases best known for their role in anchoring proteins to the cell wall in gram-positive bacteria (45). Although bioinformatic analyses have predicted the presence of sortase homologs (marine sortases) in some gram-negative bacteria, including *Shewanella* species, their biological functions have remained largely uncharacterized. Here, we provide the first experimental evidence linking a predicted gram-negative sortase system to a specific physiological function, antibiotic tolerance. In *S. oneidensis*, the gene *SO2195*, which is annotated as encoding an inter-alpha-trypsin inhibitor family protein, contains a canonical PQTST motif predicted to be recognized by the marine sortase SO2196 (46).

Mechanistically, SO2195 does not act as a β-lactamase or antibiotic-sequestering protein, as neither MICs nor β-lactamase activity were altered upon its deletion (Fig. S7; Table S1). Instead, our data suggest a role for SO2195 in maintaining cell envelope integrity, with a pronounced effect on inner membrane permeability (Fig. 6C; Fig. S8), correlating with accelerated spheroplast swelling and lysis (Fig. 6A and B). The NPN uptake assay did not reveal a change in outer membrane permeability upon SO2195 deletion; however, given that NPN can also serve as a substrate for efflux pumps, we cannot formally exclude the possibility of subtle changes in the outer membrane. Nevertheless, the specific increase in PI uptake, together with the predicted C-terminal transmembrane domain of SO2195, raises the possibility that SO2195 may function to tether the inner membrane to the peptidoglycan layer, or following sortase-mediated cleavage, to link the peptidoglycan layer to the outer membrane. The observation that SO2195 affects cell envelope integrity aligns with the finding that in peptidoglycan-deficient cells, both the inner and outer membranes collectively withstand turgor pressure to maintain spheroplast viability (47). The contribution of SO2195 extended to multiple β-lactam classes, including carbapenems and cephalosporins (Fig. 4D), as well as to D-cycloserine, suggesting a general role in envelope stress response. However, heterologous overexpression of *SO2195* in *E. coli* K-12 did not prevent antibiotic-induced lysis (Fig. S9), indicating that SO2195 alone is insufficient to confer tolerance under these conditions. Whether SO2195 requires the cognate sortase SO2196 or acts independently remains to be determined. The conserved synteny of SO2195 and SO2196 suggests a functional linkage (Fig. 7), but the precise nature of this interaction awaits future investigation.

Notably, SO2195 homologs are widespread across Proteobacteria and several other phyla, and their genes are almost always located adjacent to a sortase gene, forming a conserved genetic module. This module is highly enriched in genera, such as *Shewanella* and *Bradyrhizobium*, which have recently been reported to survive exposure to CWA antibiotics (48, 49), suggesting that it may represent a broadly used cell wall stress response system. Nevertheless, in a typical gram-positive sortase system, the cleaved substrate is covalently linked to the pentaglycine cross-bridge of peptidoglycan. The cell wall architecture of gram-negative bacteria differs fundamentally from that of gram-positive bacteria, as it possesses an outer membrane and lacks the pentaglycine bridge in its peptidoglycan layer. Therefore, the ultimate anchoring site of SO2195 after cleavage remains unknown. Elucidating this question will be important for understanding how sortase-substrate systems function in gram-negative bacteria.

While the molecular basis of antibiotic tolerance remains less understood than resistance, accumulating evidence indicates that tolerance can accelerate the evolution of stable resistance in diverse bacteria (5, 6, 8, 10). Our findings provide a mechanistic explanation for this model. We show that in *S. oneidensis*, a single regulatory system, PghKR, coordinately controls both antibiotic tolerance and resistance. This integrated defense strategy may offer an evolutionary advantage, as tolerance can sustain a viable bacterial population during transient antibiotic exposure, thereby increasing the opportunity for the subsequent acquisition or selection of resistance mutations. Importantly, the coexistence of both survival strategies within a single organism implies that clinically relevant, high-level resistant pathogens may also harbor cryptic tolerance mechanisms, potentially compromising therapies that target resistance alone. By elucidating how a bacterium dynamically expresses resistance and tolerance, this work highlights the importance of targeting both phenotypes to effectively overcome antibiotic treatment failure.

## MATERIALS AND METHODS

### Bacterial strains, plasmids, and culture conditions

All bacterial strains and plasmids used in this study are listed in Table S2. *S. oneidensis* and *E. coli* strains were routinely grown in lysogeny broth (LB, Difco, Detroit, MI) or agar at 30°C and 37°C, respectively. When needed, the medium was supplemented with chemicals at the following concentrations: gentamicin (Gm), 50 μg/mL; kanamycin (Km), 50 μg/mL; and 2,6-diaminopimelic acid (DAP), 0.3 mmol/L. For tolerance assays, the following concentrations were used: 20 μg/mL ampicillin (AMP), 256 μg/mL carbenicillin (Carb), 120 μg/mL D-cycloserine (DCS), 256 μg/mL meropenem, and 128 μg/mL cefalexin. The multiples of the MIC that these concentrations represent for each strain are provided in Table S1. To induce the P_BAD_ promoter, L-arabinose was added to the culture medium at a final concentration of 0.02% (wt/vol); any deviations from this concentration are noted in the text.

### In-frame deletion mutagenesis and complementation

In-frame deletion mutants of *S. oneidensis* were generated using an *att*-based fusion PCR strategy, as previously reported (50). Briefly, two DNA fragments flanking the target gene were amplified using primer pairs 5O/5I and 3O/3I, and subsequently fused via overlap-extension PCR. The resulting fusion fragment was cloned into the suicide vector pHGM01 through a BP recombination reaction with Gateway BP Clonase II (Invitrogen) following the manufacturer’s protocol. These constructs were first maintained in *E. coli* WM3064 (a DAP auxotroph) and then transferred into the appropriate *S. oneidensis* strain by conjugation. Chromosomal integration of the deletion construct was selected based on gentamycin resistance and sucrose sensitivity, and confirmed by PCR. Verified transconjugants were cultured in LB without NaCl and plated on LB containing 10% (wt/vol) sucrose. Colonies that were gentamycin-sensitive and sucrose-resistant were screened by PCR to confirm the deletion of the target gene, and successful mutants were further validated by DNA sequencing.

The arabinose-induced plasmid pHG-P_BAD_ harboring the corresponding gene was used for genetic complementation (51), such as P_BAD_-*pdsO*, P_BAD_-*SO2195*, and P_BAD_-*SO2196*. After sequencing verification, the vectors were transferred into the deletion mutants by conjugation. All primers used in this study are listed in Table S3.

### Time-dependent killing experiments and regrowth of *S. oneidensis*

Overnight cultures of *S. oneidensis* and *E. coli* strains were diluted 1:100 into 3 mL fresh LB broth and were grown to the exponential phase (OD_600_ of ~0.1). Various concentrations of ampicillin, carbenicillin, D-cycloserine, meropenem, or cefalexin were added. At each time point, samples were washed with fresh LB and serially diluted 10-fold in fresh LB broth. Ten microliters of each dilution was plated onto LB agar. Viable cell counts (CFU per milliliter) were counted after 16 h of growth at 30°C.

For regrowth of *S. oneidensis*, after 2 h of treatment with 20 μg/mL ampicillin, samples were washed, resuspended in fresh LB broth with or without ampicillin, and then incubated at 30°C with 200 rpm shaking for 6 h. The CFU/mL was measured every hour.

### Phase-contrast microscopy

Overnight cultures of the *S. oneidensis* and *E. coli* strains were diluted 1:100 into 3 mL fresh LB broth and were grown to the exponential phase (OD_600_ of ~0.1). Various concentrations of antibiotics were added. At each time point, cells were fixed on a glass slide and visualized with a Motic BA410E phase-contrast microscope. Micrographs were captured with a Moticam Pro 285A digital camera and Motic images advanced 3.2 software (Motic Incorporation, China). Cell area was measured with ImageJ software.

### Total RNA extraction and quantitative real-time reverse transcription PCR

Quantitative real-time reverse transcription PCR (qRT-PCR) was performed as described previously (52). Strains were incubated at 30°C for the appropriate time and harvested. Total RNA was extracted using RNAiso Plus* (Takara, Beijing, China). The depletion of genomic DNA and the synthesis of cDNA were processed using HiScript III RT SuperMix for qPCR (+gDNA wiper) (Vazyme, Nanjing, China). Three independent qRT-PCR experiments were performed using ChamQ Universal SYBR qPCR Master Mix (Vazyme, Nanjing, China). The transcript level of each gene was normalized by 16S rRNA in each sample using the CT (2^−∆∆CT^) method.

### Propidium iodide uptake assay

Bacterial strains were treated with antibiotics under the same conditions as described for the time-killing curve assay. After 6 h of treatment, the cultures were harvested, washed with PBS, and resuspended to an OD_450_ of 0.4–0.5. The cell suspensions were then incubated with PI in the dark for 30 min. Fluorescence was measured at an excitation wavelength of 535 nm and an emission wavelength of 615 nm. The final fluorescence value for each strain was calculated as the ratio of the measured fluorescence to the OD_450_ of the corresponding sample.

### Bioinformatic analysis of SO2195 homologs

To identify homologs of SO2195, PSI-BLAST searches were performed against the NCBI ClusteredNR database using the SO2195 amino acid sequence as a query, with an E-value threshold of 0.05 (53). A total of 1,000 homologous sequences were retrieved. Multiple sequence alignment was conducted using MAFFT with the --auto option and eight threads (54). The resulting alignment was trimmed using trimAl with the -automated1 parameter to remove poorly aligned and gap-rich regions (55). Maximum likelihood phylogenetic analysis was performed using IQ-TREE2 (56). The best-fit substitution model was automatically selected by ModelFinder Plus (-m MFP), and branch support was assessed with 1,000 ultrafast bootstrap replicates. The tree was visualized and annotated using the iTOL online tool (57). The protein accessions of the identified homologs were submitted to the webFlaGs webserver (https://server.atkinson-lab.com/webflags). Using default parameters, the tool generated a to-scale gene arrangement diagram where conserved flanking genes are clustered and color-coded (58).

### Statistical analyses

Experiments were carried out with at least three biological replicates. Data are shown as means from replicate experiments, with error bars representing standard deviation. Unless otherwise indicated, Student’s *t*-test was used for comparisons between two groups. Statistical significance is denoted as follows: ns, not significant; *P < 0.05; **P < 0.01; ***P < 0.001; ****P < 0.0001.

## SUPPLEMENTAL MATERIAL

Supplemental material mbio.01515-26-s0001.pdf Fig. S1 to S9; Tables S1 to S3.

ASM does not own the copyrights to Supplemental Material that may be linked to, or accessed through, an article. The authors have granted ASM a non-exclusive, world-wide license to publish the Supplemental Material files. Please contact the corresponding author directly for reuse.
