# Supplementary material for: Shewanella oneidensis employs both β-lactam resistance and tolerance controlled by the PghKR system
Source: mBio. 2026 Sep 14;17(10):e01515-26. doi: 10.1128/mbio.01515-26 (PMC13712847; doi:10.1128/mbio.01515-26)
Supplement: Supplementary file 1 [file mbio.01515-26-s0001.pdf]

Supplemental Material for

Shewanella oneidensis employs both β-lactam resistance and tolerance controlled by the PghKR system

Jingxiao Cai[1], Yanqun Liang[1], Yu Chen[1], Xinyu Weng[1], Yiwei Hu[1], Min Wang[1], Qiu Meng[1], Zhiliang Yu[1], Jianhua Yin[1,2*]

1. College of Biotechnology and Bioengineering, Zhejiang University of Technology, Hangzhou 310014, China
2. State Key Laboratory of Green Chemical Synthesis and Conversion, Zhejiang University of Technology, Huzhou 313099, China

[*]For correspondence: jianhuay@zjut.edu.cn (J.Y.)

Running title: β-lactam resistance and tolerance in *Shewanella*.

Methods

Growth curve determination. Overnight cultures were diluted 1:100 into fresh LB medium and incubated at 30°C. OD_{600} was measured every hour.

β-Lactamase activities assay. Nitrocefin hydrolysis was used to assess β-lactamase activity following a previously described protocol (1). Briefly, bacterial cultures were grown to an OD_{600} of ~0.4, and cell pellets were collected by centrifugation, washed with phosphate-buffered saline (PBS), and disrupted by sonication. The resulting crude lysates were normalized for protein content (34 µg per reaction) using the Bradford assay (Beyotime, Shanghai, China). Each reaction contained 8 µg of nitrocefin (Shanghai yuanye Bio-Technology Co., Ltd., Shanghai, China), and the increase in absorbance at 486 nm was monitored.

1-N-Phenylnaphthylamine (NPN) uptake assay. The hydrophobic fluorescent probe NPN was used to assess outer membrane permeability as described (2). Briefly, cells treated with antibiotics for 6 hours were harvested by centrifugation, washed once with PBS, and resuspended to an OD_{450} of 0.4-0.5. The cell suspension was then incubated with 0.05 mM NPN for 30 min. Fluorescence was measured at excitation and emission wavelengths of 355 nm and 402 nm, respectively. The final fluorescence value for each strain was calculated as the ratio of the measured fluorescence to the OD_{450} of the corresponding sample.

Determination of MIC. The MICs of antibiotics were determined using the broth microdilution method. Briefly, overnight cultures were diluted 1:100 into fresh LB medium and grown to mid-exponential phase (OD_{600} of ~0.5). Cultures were then diluted to approximately 5×10^5 CFU/mL in LB. Twofold serial dilutions of antibiotics were prepared in 96-well microtiter plates, and 100 µL of the bacterial suspension was added to each well to a final volume of 200

µL. Plates were incubated at 30°C for *S. oneidensis* or 37°C for *E. coli* for 24 h with 200 rpm shaking. The MIC was defined as the lowest antibiotic concentration that completely inhibited visible bacterial growth. Each assay was performed with at least three independent biological replicates.

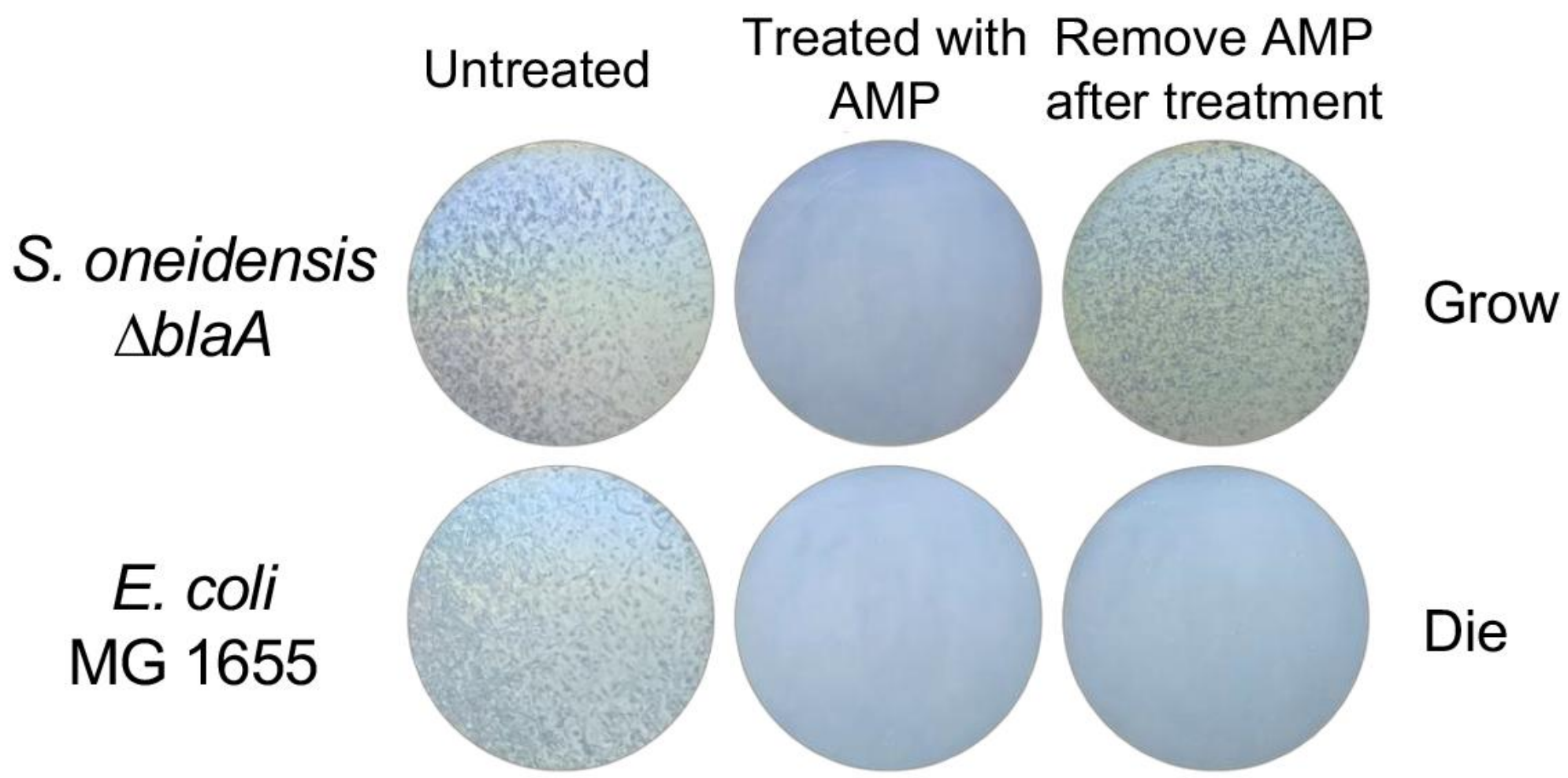

Fig. S1. Growth of *S. oneidensis* ∆*blaA* and *E. coli* MG1655 on LB plates. Left, untreated control; Middle, cells treated with 50 μg/mL ampicillin (AMP) for 2 h; Right, cells exposed to 50 μg/mL AMP for 2 h, followed by removal of the antibiotic through washing. *S. oneidensis* Δ*blaA*, but not *E. coli* MG1655, resumed growth after antibiotic removal.

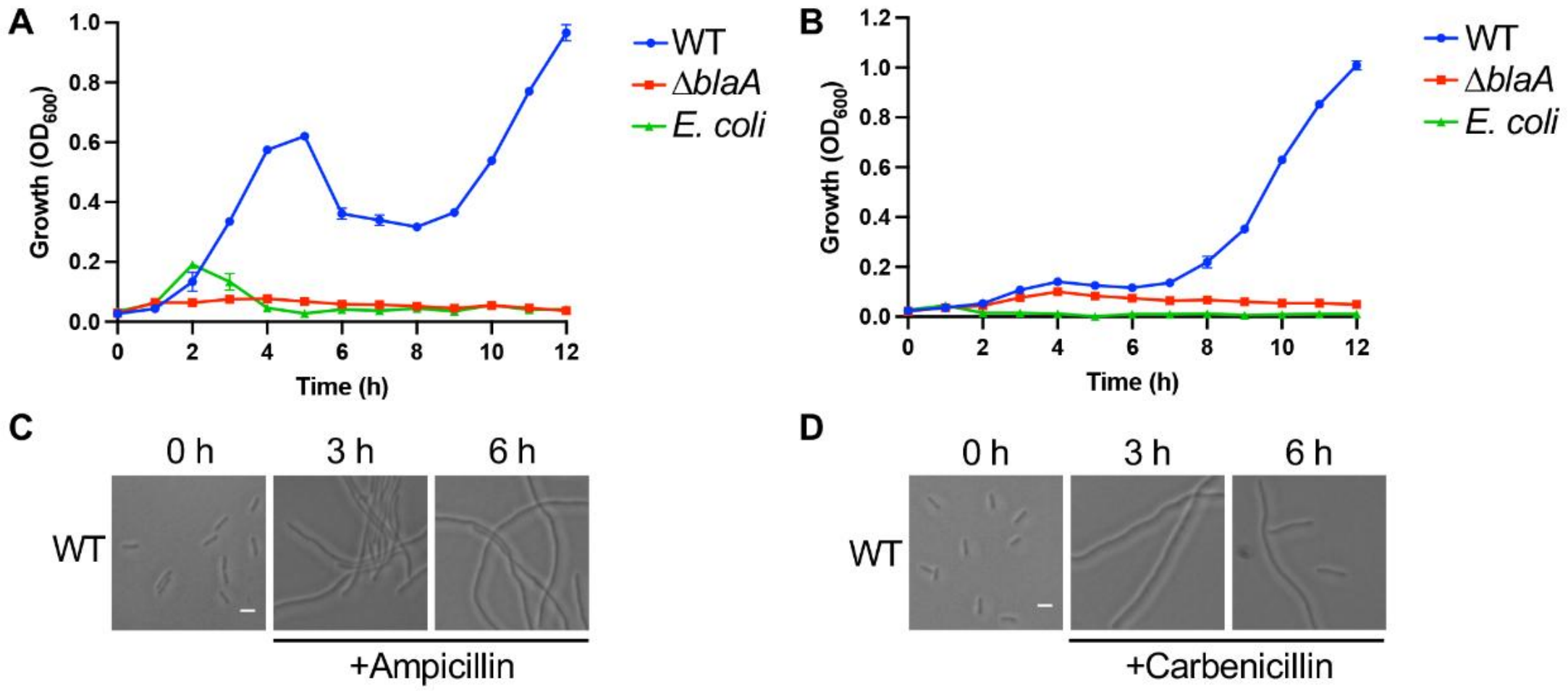

Fig. S2. Growth curve and morphology of *S. oneidensis* WT to β-lactams. (A, B) Growth curves of WT in the presence of 20 μg/mL ampicillin (A) or 256 μg/mL carbenicillin (B). Antibiotics were added immediately upon transfer of overnight cultures to fresh LB medium. **(C, D)** Morphology of WT cells after treatment with 20 μg/mL ampicillin (C) or 256 μg/mL carbenicillin (D) Antibiotics were added when the cultures reached an OD_{600} of 0.1. Scale bar, 2.5 μm.

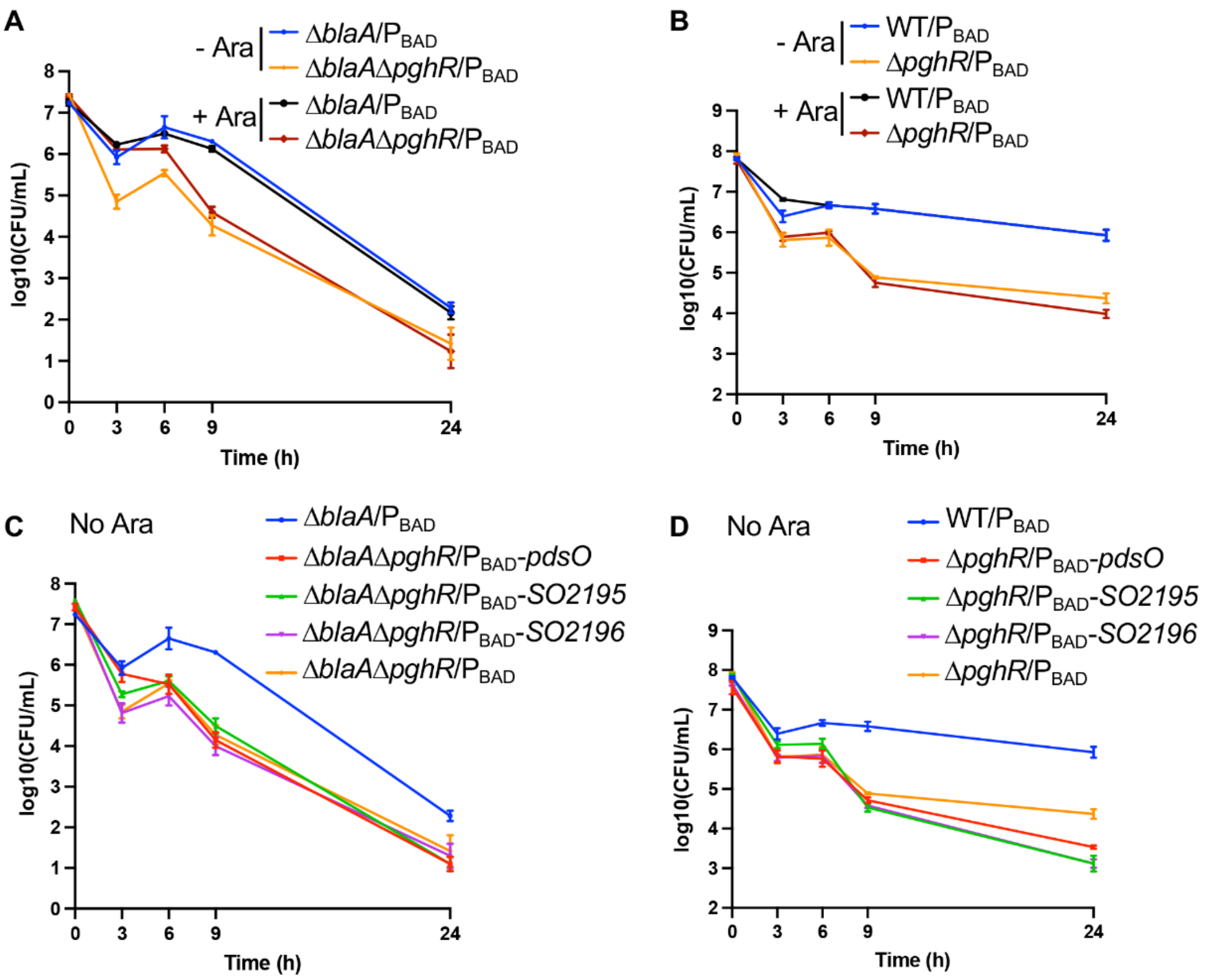

Fig. S3. Control experiments for complementation. (A, B) In the presence of 256 µg/mL carbenicillin (A) or 120 µg/mL D-cycloserine (B), the addition of arabinose and the empty vector pHG-P$_{BAD}$ (empty vector) did not affect bacterial survival. **(C, D)** Under carbenicillin (C) or D-cycloserine (D) stress, basal (uninduced) expression of genes from the P$_{BAD}$ promoter did not increase the viable cell count of the PghR-deleted mutant. Data are presented as mean ± S.D. (*n* = 3), with bacterial survival shown as $\log_{10}$(CFU/mL).

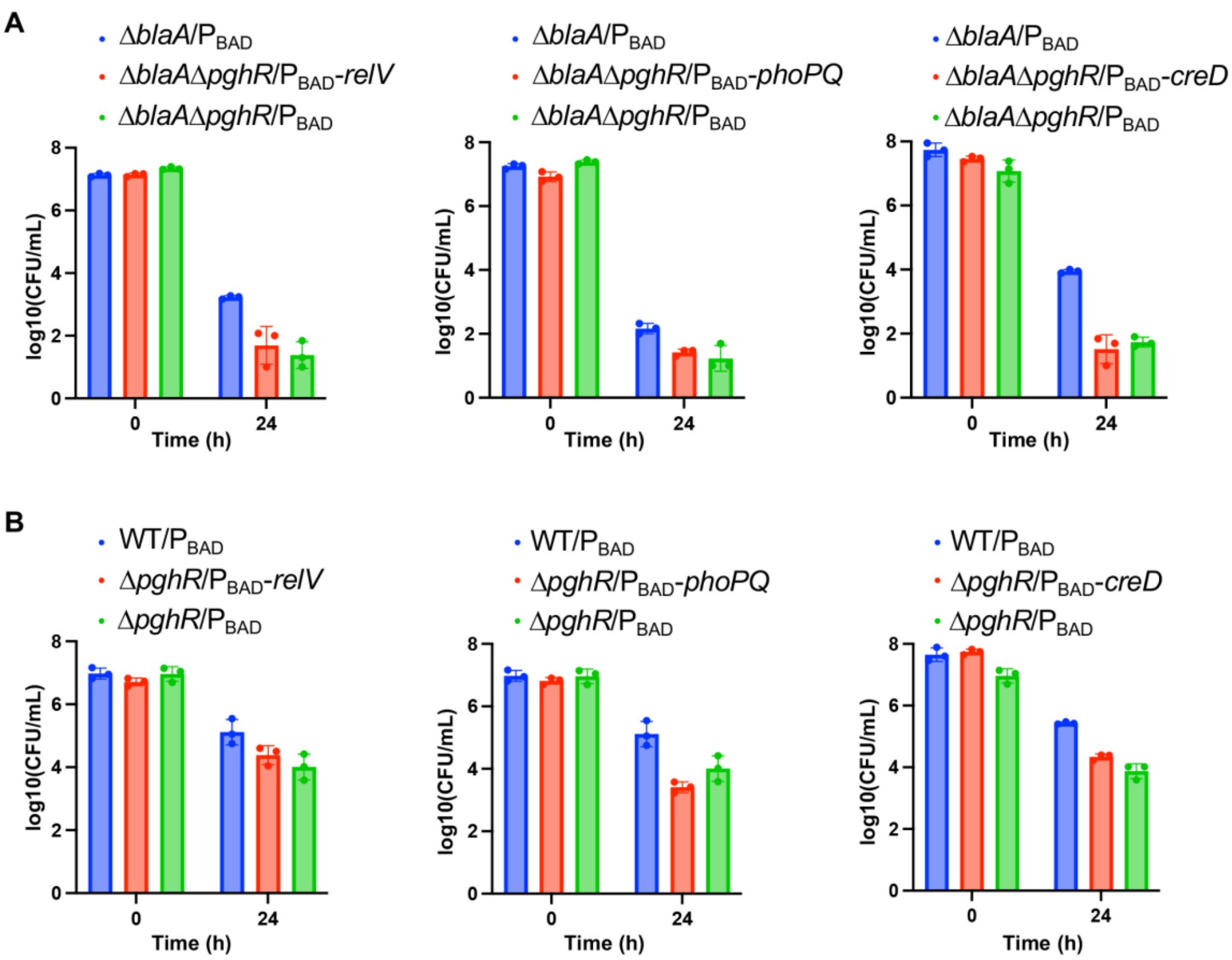

Fig. S4. Effects of PghR regulon members on antibiotic tolerance. In the presence of 256 μg/mL carbenicillin **(A)** or 120 μg/mL D-cycloserine **(B)**, the indicated PghR-regulated genes were induced with 0.02% (w/v) arabinose, and viable cell counts were determined. Data are presented as mean ± S.D. (n = 3), with bacterial survival shown as $\log_{10}$(CFU/mL).

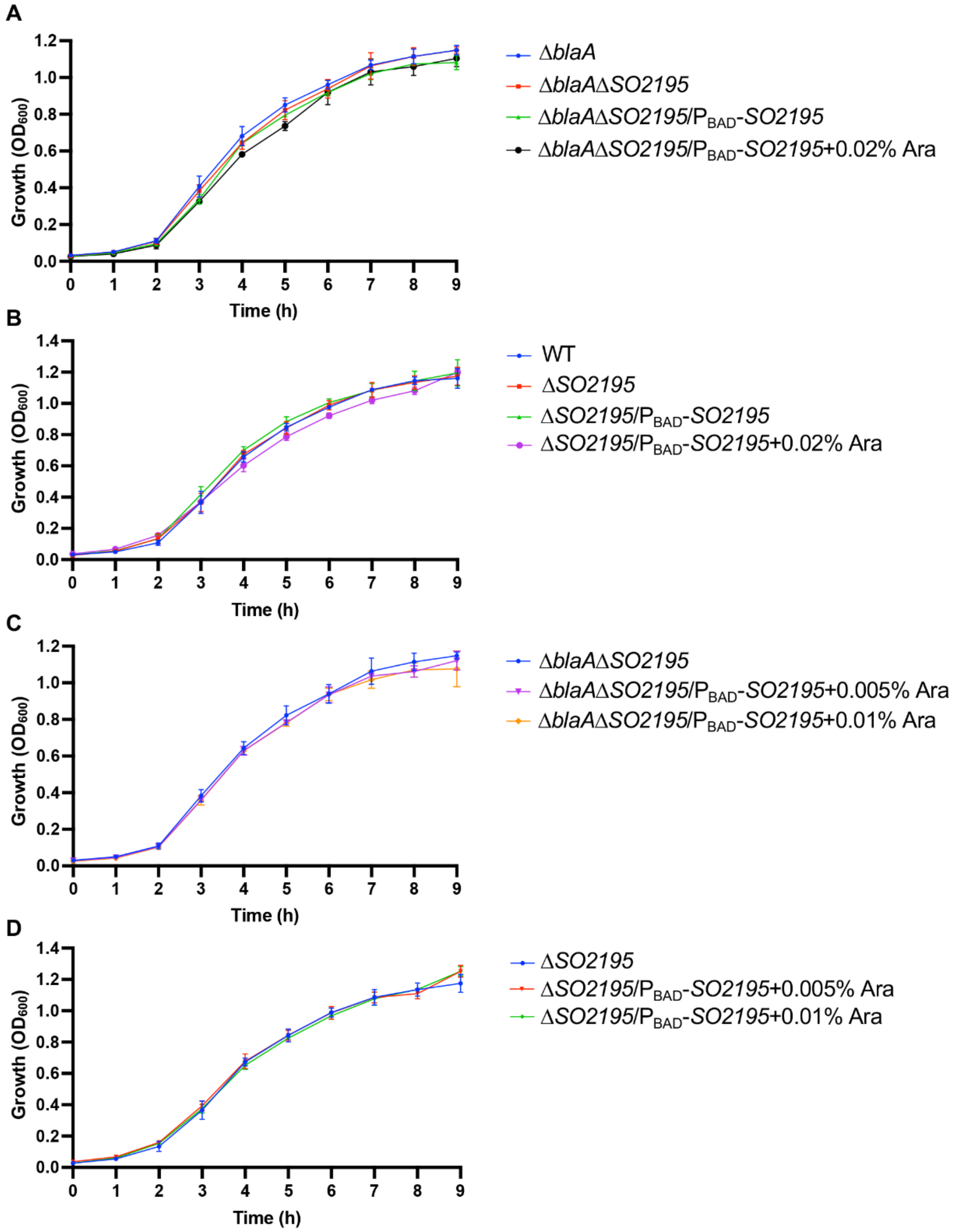

Fig. S5. Effect of arabinose concentration on bacterial growth. (A, B) Induction of *SO2195* expression with 0.02% (w/v) arabinose slightly inhibited growth. **(C, D)** Effects of different arabinose concentrations on the strain expressing SO2195. 0.01% (w/v) arabinose did not affect growth (used for complementation assays). Data are presented as mean ± S.D. (n = 3).

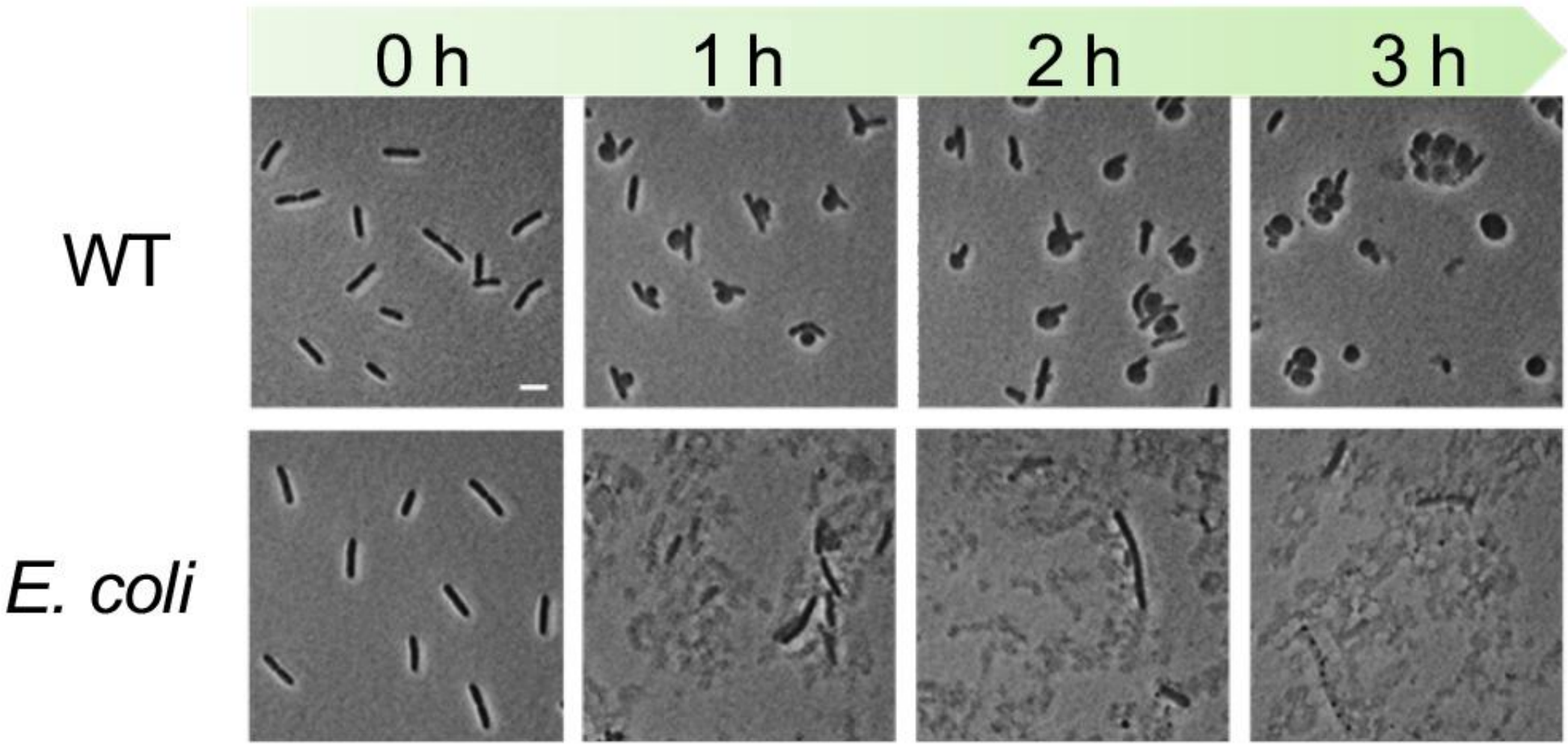

Fig. S6. Cell morphology of *S. oneidensis* and *E. coli* MG1655 upon exposure to 120 µg/mL D-cycloserine. Antibiotics were added when the cultures reached an OD_{600} of 0.1. Scale bar, 2.5 µm.

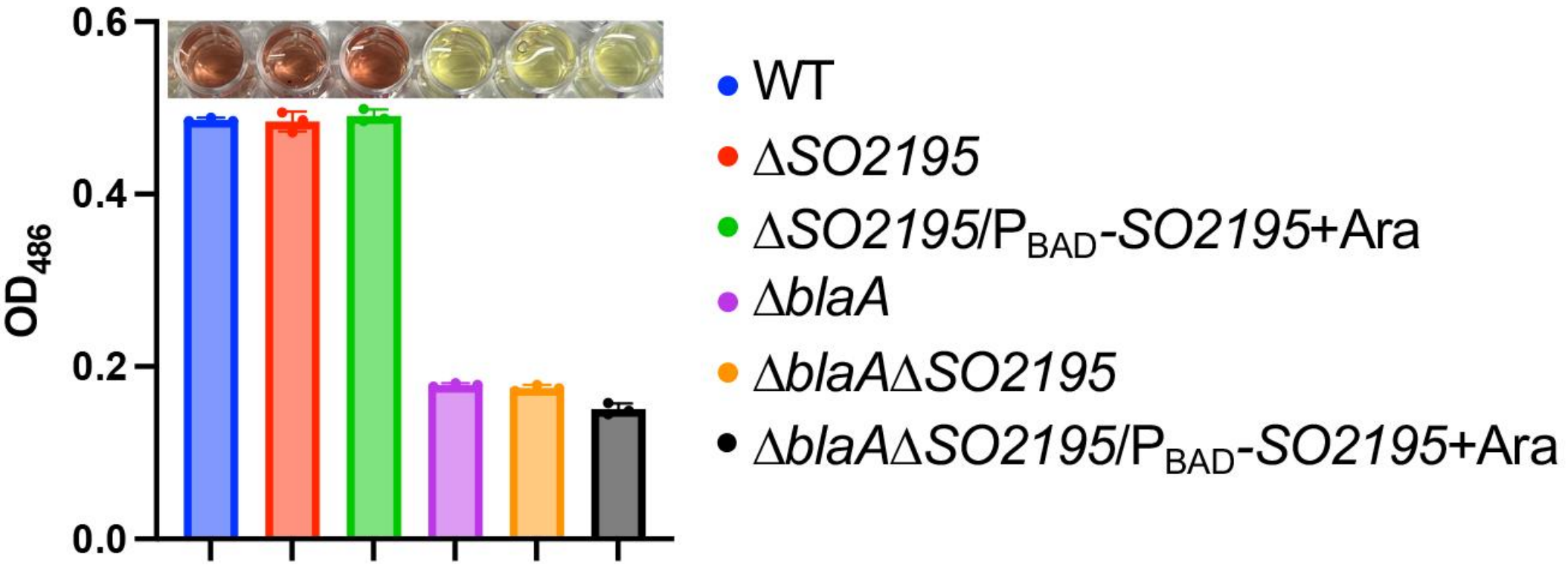

Fig. S7. β-Lactamase activities of the indicated strains. Where indicated, 0.01% (w/v) arabinose was added for induction. Cells were harvested at an OD_{600} of ~0.4, washed with PBS, and disrupted by sonication. Crude lysates were normalized to 34 µg of protein per reaction and incubated with nitrocefin. Hydrolysis was monitored by measuring the increase in absorbance at 486 nm. Data are presented as mean ± S.D. (n = 3).

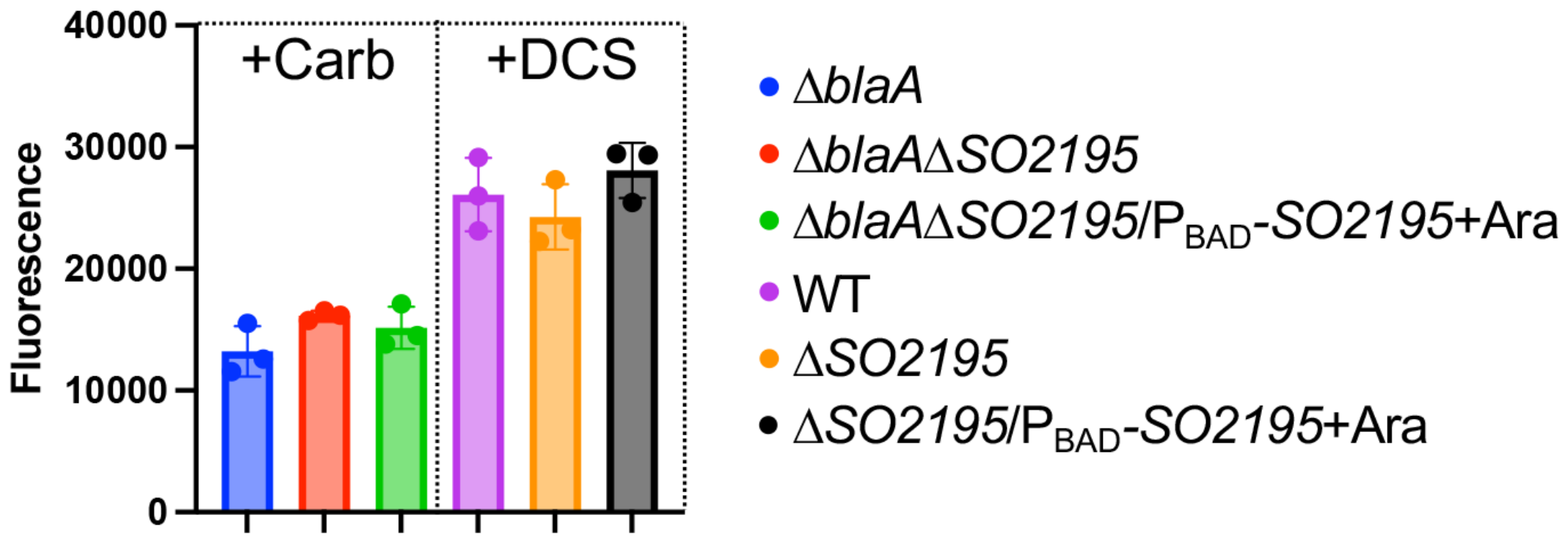

Fig. S8. NPN fluorescence after 6 h of antibiotic treatment. Cells were treated with 256 μg/mL carbenicillin or 120 μg/mL D-cycloserine. Where indicated, 0.01% (w/v) arabinose was added for induction. After antibiotic treatment, cells were harvested, washed with PBS, resuspended to an OD_{450} of 0.4-0.5, and incubated with 0.05 mM NPN for 30 min. Fluorescence (ex/em = 355/402 nm) was normalized to OD_{450}. Data are presented as mean ± S.D. (n = 3).

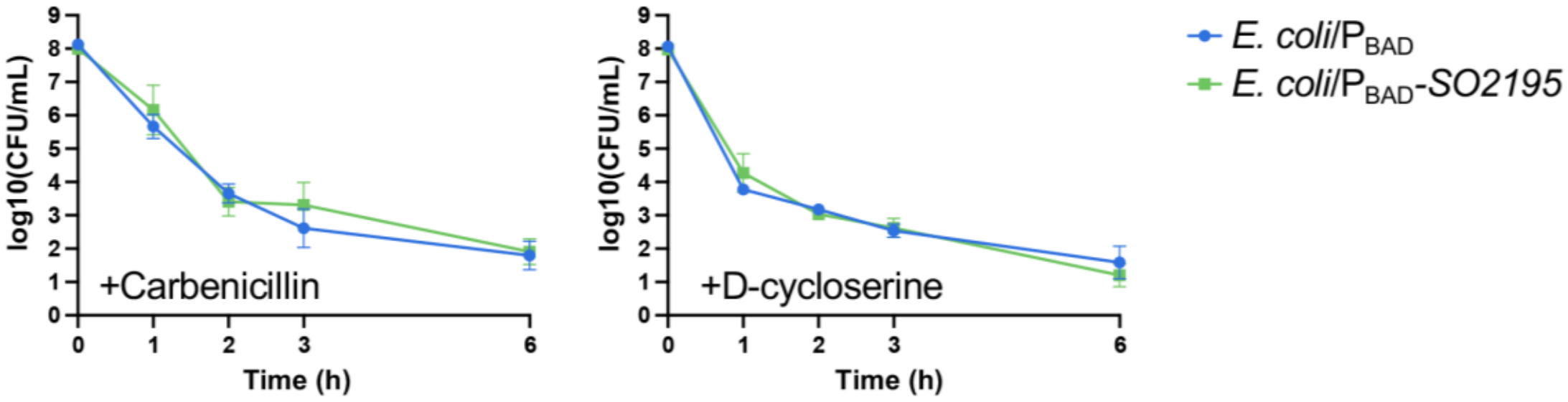

Fig. S9. Survival of *E. coli* overexpressing *SO2195* upon antibiotic treatment. When the OD_{600} reached 0.1, antibiotics were added and CFU/mL was determined at the indicated time points. Arabinose was added at 0.02% (w/v). Data are presented as mean ± S.D. (*n* = 3).

Supplement Tables

Table S1. MICs and fold-MIC values of antibiotics used in this study.

Antibiotics	Strains	MIC (μg/mL)	Used concentration (Fold-MIC; μg/mL)
Ampicillin	WT	4	20 (5 ×)
	Δ*blaA*	0.5	20 (40 ×)
	E. coli MG1655	16	20 (1.25 ×)
Carbenicillin	WT	128	256 (2 ×)
	Δ*blaA*	< 0.125	256 (> 2048 ×)
	E. coli MG1655	8	256 (32 ×)
	Δ*blaA*Δ*pghR*	< 0.125	256 (> 2048 ×)
	Δ*blaA*Δ*pghR*/*pghR*	< 0.125	256 (> 2048 ×)
	Δ*blaA*Δ*pghR*/$pghR^{D52A}$	< 0.125	256 (> 2048 ×)
	Δ*blaA*Δ*SO2195*	< 0.125	256 (> 2048 ×)
	Δ*blaA*Δ*SO2195*/P_{BAD}-*SO2195*	< 0.125	256 (> 2048 ×)
D-cycloserine	WT	32	120 (3.75 ×)
	Δ*pghR*	32	120 (3.75 ×)
	Δ*pghR*/*pghR*	32	120 (3.75 ×)
	Δ*pghR*/$pghR^{D52A}$	32	120 (3.75 ×)
	E. coli MG1655	64	120 (1.875×)
	Δ*SO2195*	32	120 (3.75 ×)
	Δ*SO2195*/P_{BAD}-*SO2195*	32	120 (3.75 ×)
Meropenem	WT	16	256 (16 ×)
	Δ*blaA*	< 0.125	256 (> 2048 ×)
	Δ*blaA*Δ*SO2195*	< 0.125	256 (> 2048 ×)
	Δ*blaA*Δ*SO2195*/P_{BAD}-*SO2195*	< 0.125	256 (> 2048 ×)
Cefalexin	WT	16	128 (8 ×)
	Δ*blaA*	16	128 (8 ×)
	Δ*blaA*Δ*SO2195*	16	128 (8 ×)
	Δ*blaA*Δ*SO2195*/P_{BAD}-*SO2195*	16	128 (8 ×)

Table S2. Strains and plasmids used in this study.

Strains or plasmids	Description	Source/ Reference
***E. coli* strains**		
DH5α	Host strain for plasmids	Lab stock
WM3064	Donor strain for conjugation; Δ*dapA*	W. Metcalf, UIUC
MG1655	Wild-type	Coli Genetic Stock Center
E. coli/P_{BAD}	Wild-type MG1655 harboring pHG-P_{BAD}	This study
E. coli/P_{BAD}-*SO2195*	Wild-type MG1655 harboring pHG-P_{BAD}-*SO2195*	This study
***S. oneidensis* strains**		
MR-1	Wild-type	Lab stock
Δ*blaA*	In-frame deletion in *blaA*	Yin et al. 2013
Δ*pghR*	In-frame deletion in *pghR*	Yin et al. 2023
Δ*blaA*Δ*pghR*	In-frame deletion in *blaA* and *pghR*	This study
Δ*blaA*Δ*pghR*/*pghR*	Δ*blaA*Δ*pghR* harboring pHG101-*pghR*	This study
Δ*blaA*Δ*pghR*/*pghR*^{D52A}	Δ*blaA*Δ*pghR* harboring pHG101-*pghR*^{D52A}	This study
Δ*blaA*/P_{BAD}	Δ*blaA* harboring pHG-P_{BAD}	This study
Δ*blaA*Δ*pghR*/P_{BAD}-*pdsO*	Δ*blaA*Δ*pghR* harboring pHG-P_{BAD}-*pdsO*	This study
Δ*blaA*Δ*pghR*/P_{BAD}-*SO2195*	Δ*blaA*Δ*pghR* harboring pHG-P_{BAD}-*SO2195*	This study
Δ*blaA*Δ*pghR*/P_{BAD}-*SO2196*	Δ*blaA*Δ*pghR* harboring pHG-P_{BAD}-*SO2196*	This study
Δ*blaA*Δ*pghR*/P_{BAD}-*creD*	Δ*blaA*Δ*pghR* harboring pHG-P_{BAD}-*creD*	This study
Δ*blaA*Δ*pghR*/P_{BAD}-*relV*	Δ*blaA*Δ*pghR* harboring pHG-P_{BAD}-*relV*	This study
Δ*blaA*Δ*pghR*/P_{BAD}-*phoPQ*	Δ*blaA*Δ*pghR* harboring pHG-P_{BAD}-*phoPQ*	This study
Δ*blaA*Δ*pghR*/P_{BAD}	Δ*blaA*Δ*pghR* harboring pHG-P_{BAD}	This study
Δ*blaA*Δ*SO2195*	In-frame deletion in *blaA* and *SO2195*	This study
Δ*blaA*Δ*SO2195*/P_{BAD}-*SO2195*	Δ*blaA*Δ*SO2195* harboring pHG-P_{BAD}-*SO2195*	This study
Δ*pghR*/*pghR*	Δ*pghR* harboring pHG101-*pghR*	Yin et al. 2023
Δ*pghR*/*pghR*^{D52A}	Δ*pghR* harboring pHG101-*pghR*^{D52A}	Yin et al. 2023
WT/P_{BAD}	MR-1 harboring pHG-P_{BAD}	This study
Δ*pghR*/P_{BAD}-*pdsO*	Δ*pghR* harboring pHG-P_{BAD}-*pdsO*	This study
Δ*pghR*/P_{BAD}-*SO2195*	Δ*pghR* harboring pHG-P_{BAD}-*SO2195*	This study

Δ*pghR*/P_{BAD}-*SO2196*	Δ*pghR* harboring pHG-P_{BAD}-*SO2196*	This study
Δ*pghR*/P_{BAD}-*creD*	Δ*pghR* harboring pHG-P_{BAD}-*creD*	This study
Δ*pghR*/P_{BAD}-*relV*	Δ*pghR* harboring pHG-P_{BAD}-*relV*	This study
Δ*pghR*/P_{BAD}-*phoPQ*	Δ*pghR* harboring pHG-P_{BAD}-*phoPQ*	This study
Δ*pghR*/P_{BAD}	Δ*pghR* harboring pHG-P_{BAD}	This study
Δ*SO2195*	Δ*SO2195* derived from MR-1	This study
Δ*SO2195*/P_{BAD}-*SO2195*	Δ*SO2195* harboring pHG-P_{BAD}-*SO2195*	This study
**Plasmids**		
pHGM01	Ap^r, Gm^r, Cm^r, suicide vector	Jin et al. 2013
pHGM01-*blaA*	pHGM01 carrying the upstream and downstream of *blaA*	Jin et al. 2013
pHGM01-*SO2195*	pHGM01 carrying the upstream and downstream of *SO2195*	This study
pHG101	Km^r, promoterless vector for complementation	Wu et al. 2011
pHG101-*pghR*	Expression of *pghR* controlled by its native promoter (P_{pgh})	Yin et al. 2023
pHG101-*pghR*^{D52A}	Expression of *pghR*^{D52A} controlled by the P_{pgh} promoter	Yin et al. 2023
pHG-P_{BAD}	Km^r, arabinose-induced expressing vector for complementation	Yin et al. 2015
pHG-P_{BAD}-*pdsO*	Expression of *pdsO* controlled by the P_{BAD} promoter	This study
pHG-P_{BAD}-*SO2195*	Expression of *SO2195* controlled by the P_{BAD} promoter	This study
pHG-P_{BAD}-*SO2196*	Expression of *SO2196* controlled by the P_{BAD} promoter	This study
pHG-P_{BAD}-*relV*	Expression of *relV* controlled by the P_{BAD} promoter	This study
pHG-P_{BAD}-*creD*	Expression of *creD* controlled by the P_{BAD} promoter	This study
pHG-P_{BAD}-*phoPQ*	Expression of *phoPQ* controlled by the P_{BAD} promoter	This study

Table S3. Primers used in this study.

Primer name	Sequence (5′→3′)
**In-frame gene deletion**	
SO2195-5O	GGGGACAAGTTTGTACAAAAAAGCAGGCTTAATTCAACAGGGCGTTGCA
SO2195-5I	GATTCATCTACATGCTGAGGCCTGATACCCGCATCGA
SO2195-3I	CTCAGCATGTAGATGAATCTGAGTCGTGATGCTGTGGTT
SO2195-3O	GGGGACCACTTTGTACAAGAAAGCTGGGTTGACGCACTCGACAACACAA
SO2195-LF	GAGCCACAAACTGAATGGAC
SO2195-LR	CACCACGAAACGCAGTTCAG
SO2195-SF	CAACTGGCTAGCGAGCAAC
SO2195-SR	TCAATGGCTAAGGGCCTG
**Genetic complementation**	
P_{BAD}-*pdsO*-F	TGGGGATCCGAGCTCGAGATGAAAAAGACGCTAATCAGTG
P_{BAD}-*pdsO*-R	AACAGCCAAGCTTCGAATTCTTATTCTGTTACGCGGCTC
P_{BAD}-*SO2195*-F	TGGGGATCCGAGCTCGAGATGATCACAAAGAACAGGGT
P_{BAD}-*SO2195*-R	AACAGCCAAGCTTCGAATTCTCAATGGCTAAGGGCCTG
P_{BAD}-*SO2196*-F	TGGGGATCCGAGCTCGAGATGACAACAAAGCAGATACAGAT
P_{BAD}-*SO2196*-R	AACAGCCAAGCTTCGAATTCTTATGTAGAATAACTACACAGCACG
P_{BAD}-*phoPQ*-F	TGGGGATCCGAGCTCGAGATGAGAATTTTGGTTGTTGAAGATG
P_{BAD}-*phoPQ*-R	AACAGCCAAGCTTCGAATTCTCAGGCAGGGATGGTAATCC
P_{BAD}-*relV*-F	TGGGGATCCGAGCTCGAGATGAATAAGTTATTTCGTACCTTTTTTATC
P_{BAD}-*relV*-R	AACAGCCAAGCTTCGTTAAGCGACTGAGCTGTTTAGC
P_{BAD}-*creD*-F	TGGGGATCCGAGCTCGAGATGTTATTCAGCATTATTTTCGGGT
P_{BAD}-*creD*-R	AACAGCCAAGCTTCGAATTCCTATTGCGCCGTATTCTTGG
**qRT-PCR**	
qRT-*pdsO*-F	GAGCCACAAACTGAATGGAC
qRT-*pdsO*-R	AAGGTCCCAGTAATACCACCG
qRT-*SO2195*-F	CGCAGAATATCACCGCCGTA
qRT-*SO2195*-R	CATCGAGACTTGGGTGTCCA
qRT-*SO2196*-F	AAGTGGTGGCGACTAAGGTG
qRT-*SO2196*-R	CACCACGAAACGCAGTTCAG
qRT-*pghR*-F	CCTACGTGCCATGTCAAACAC
qRT-*pghR*-R	GATTTGCGTCGATGGTGAGTG
qRT-16S-F	TGATGTCGCAAGTTCAGTCG
qRT-16S-R	GCCGAAATAGCAACGCAGTT

Reference

1. Li L, Wang Q, Zhang H, Yang M, Khan MI, Zhou X. 2016. Sensor histidine kinase is a β-lactam receptor and induces resistance to β-lactam antibiotics. Proc Natl Acad Sci U S A 113:1648-1653.
2. Yin J, Cai J, Yuan Z, Wang Z, Gao H, Qiu J, Yu Z. 2018. Deletion of PBP1a/LpoA complex compromises cell envelope integrity in *Shewanella oneidensis*. FEMS Microbiol Lett 365.
